# Highly efficient upconversion photodynamic performance of rare-earth-coupled dual-photosensitizers: ultrafast experiments and excited-state calculations

**DOI:** 10.1515/nanoph-2023-0772

**Published:** 2024-02-05

**Authors:** Yubiao Yang, Lei Zhang, Chao Xiao, Zhencheng Huang, Fuli Zhao, Jinchang Yin

**Affiliations:** School of Physics, State Key Laboratory of Optoelectronic Materials and Technologies, Sun Yat-Sen University, Guangzhou 510275, China; School of Biomedical Engineering, Health Science Center, Shenzhen University, Shenzhen 518060, China

**Keywords:** rare-earth, photosensitizer, ultrafast dynamics, time-dependent density functional theory (TD-DFT), energy transfer, photodynamic therapy (PDT)

## Abstract

Upconversion photodynamic therapy (UC-PDT), which integrates upconversion nanoparticles (UCNPs) with photosensitizers (PSs), presents a promising advancement in the field of phototherapy. However, despite the extensive studies focused on the design and synthesis of UCNPs, there is a paucity of systematic research on the mechanisms underlying the synergistic upconversion photodynamic effects. Here we have synthesized upconversion core@dotted-shell nanoparticles (CDSNPs) and covalently tethered them with two distinct PSs, thereby constructing a dual-PS UC-PDT system with high synergistic photodynamic performance. To unravel the mechanism underlying the synergism, we employed a combination of quantum mechanical calculations and ultrafast time-resolved spectroscopy techniques. The results indicate that rare earth oxides play a pivotal role in enhancing the intersystem crossing processes of PSs through modulating their excited electronic states. Additionally, Förster resonance energy transfer between two distinct PSs contributes to the amplification of triplet state populations, thus further enhancing the photodynamic effect. *In vitro* experiments demonstrate that the prepared CDSNPs based dual-PS system exhibits excellent biocompatibility with normal cells and exceptional synergistic photodynamic efficacy against tumor cells upon near-infrared excitation. This research contributes theoretical insights into the design and application of multi-photosensitizer UC-PDT systems, laying the groundwork for more efficient preclinical implementations in the future.

## 1 Introduction

Photodynamic therapy (PDT) is a clinical treatment method that employs photosensitizers (PSs) to induce the generation of cytotoxic reactive oxygen species upon exposure to light, thereby leading to localized damage in target tissues [1], [2]. While conferring benefits such as non-invasive intervention and minimal side effects, conventional PDT is still hampered by challenges including limited tissue penetration depth, inefficient PSs, and non-targeted distribution [3], [4]. Upconversion photodynamic therapy (UC-PDT) has emerged as a promising solution to surmount these limitations and improve treatment efficacy [5], [6]. UC-PDT utilizes functional upconversion nanoparticles (UCNPs) to convert lower-energy near-infrared (NIR) light into higher-energy ultraviolet or visible luminescence for efficiently activating PSs, which enables the treatment of deeper-seated tumors upon NIR irradiation. Recent research has focused on nanoparticle design, PS loading strategies, light activation mechanisms, and has witnessed significant advancements in the field of UC-PDT [7], [8], [9], [10], [11]. In particular, lanthanide doped UCNPs have evolved into a versatile nanoplatform for both diagnosis and treatment, owing to their adaptable structure design, surface modification, and bioconjugation [12], [13]. One development is the concept of combined therapy, where UCNPs are utilized to carry various reagents, such as PSs, chemotherapy agents, and radiotherapy agents, achieving the synergistic outcomes [14], [15]. Extensive pre-clinical evidence suggests that synergistic therapy yields superior efficacies compared to single treatment paradigms [1], [16].

Mounting of distinct reagents on UCNPs reduces individual reagent dosage, thereby significantly diminishing associated side effects and mitigating long-term treatment resistance. Most importantly, this approach leverages distinct pathways of various reagents to effectively reduce the risk of cancer metastasis and recurrence. For example, Lee et al. developed a dual-PS combination system to enhance UC-PDT [17]. Chlorin e6 (Ce6) and Rose Bengal, both commonly employed PSs, were loaded on the surface of UCNPs with a core@shell structure. The upconversion photoluminescence of blue, green, and red light can be utilized to simultaneously activate different PSs when irradiated by NIR light, resulting in a significantly higher generation of reactive oxygen species compared to a single PS system. Additionally, Idris and Yang groups independently constructed a two-PS system by combining UCNPs and two PSs, merocyanine 540 (MC540), and zinc phthalocyanine [18], [19]. Their research revealed that the two-PS system induced a greater reduction in cancer cell viability in comparison to the single-PS system. Furthermore, our group previously tailored a gadolinium oxide based UCNPs with a core-satellite structure, featuring bright green, red, and NIR-II photoluminescence [20]. We covalently linked two PSs, Ce6, and MC540, to these UCNPs, and adjusted the total content of the two PSs to match that of a single-PS system. Singlet oxygen detection results indicated a marked synergistic photodynamic effect. Although previous studies have demonstrated that the dual-PS approach evidently enhances the efficacy of UC-PDT, the mechanism underlying this synergistic effect remains unclear.

In this study, MC540 and Ce6 PSs were covalently linked to Gd_2_O_3_@SiO_2_:Yb^3+^/Er^3+^/Li^+^ UCNPs with unique core@dotted-shell structure (CDSNPs) to establish a dual-PS combined therapy system. To improve biocompatibility and targeting, CDSNPs were functionalized with folate-PEG molecules. Quantum mechanical calculations based on time-dependent density functional theory (DFT) and ultrafast experiments including transient absorption (TA) spectroscopy and time-resolved fluorescence spectroscopy were employed to elucidate the potential mechanism underpinning the significantly improved UC-PDT efficacy. Simplified structural models based on practical CDSNP structures were constructed to better reveal the influence of rare earth on two PSs. CDSNPs expedite a rapid relaxation of the high-lying excited states and intersystem crossing (ISC) from singlet S_1_ to triplet T_1_ states by modulating the properties of electronically excited states of PSs. Moreover, rare earth accelerates the energy transfer from MC540 to Ce6, aligning with the Förster energy transfer mechanism. Finally, dark-toxicity and phototoxicity bioassays were conducted to validate the excellent biocompatibility and UC-PDT effectiveness in killing cancer cells. These findings have the potential to pave the way for the development of advanced UC-PDT strategies with enhanced therapeutic outcomes in cancer treatment.

## 2 Results and discussion

### 2.1 Fabrication, characterization, and optical properties

**Scheme 1:**
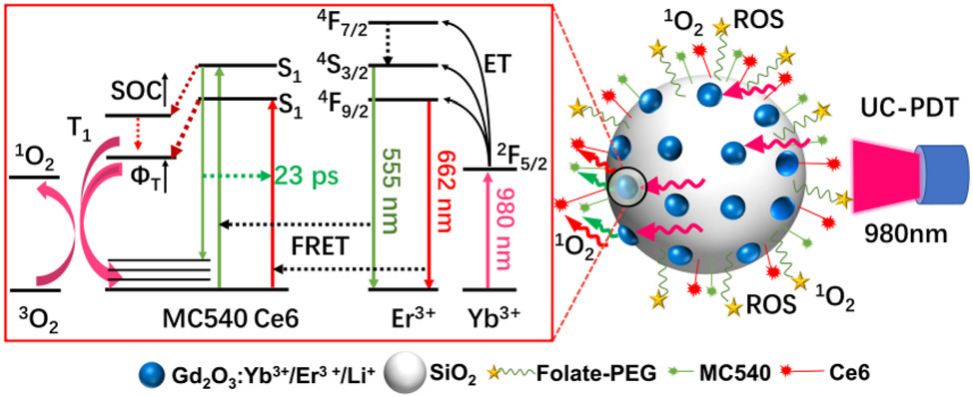
Schematic illustration of designed dual-PS UC-PDT system with an improved upconversion photodynamic performance. Here, ROS denotes reactive oxygen species, ^1^O_2_ represents singlet oxygen, ET stands for energy-transfer, FRET means Förster resonance energy transfer and SOC is spin-orbit coupling. Green dotted arrow indicates MC540 transfers energy to Ce6 within 23 ps. Dark red dotted arrow means the intersystem crossing process. Red dotted arrow reveals additional pathway where energy of T_1_ excited-state of MC540 transfers to T_1_ excited-state of Ce6.

Scheme 1 elaborates the schematic of our fabricated CDSNPs covalently conjugated with PSs, MC540, and Ce6, for boosting dual-drive upconversion photodynamic performance. Yb^3+^, Li^3+^, and Er^3+^ co-doped ultrasmall Gd_2_O_3_ NPs are embedded on the surface of silica NPs through Gd–O–Si bonds, serving as upconversion photoluminescence centers due to energy-transfer from the sensitizer Yb^3+^ to the activator Er^3+^. Specifically, Yb^3+^ is excited from the ^2^F_7/2_ energy level to the ^2^F_5/2_ energy level under 980 nm irradiation, followed by energy-transfer from Yb^3+^ to Er^3+^, especially to the ^4^F_9/2_ and ^4^S_3/2_ energy levels of Er^3+^, resulting in red and green luminescence. Förster resonance energy transfer (FRET) from CDSNPs to the two PSs is fully exploited to induce photodynamic effects, since Ce6 and MC540 linked on silica surface are in close proximity to the rare-earth doped nanocrystals, with their absorption spectra highly overlapping with the red and green emissions of CDSNPs. The ISC processes of PSs are significantly accelerated by CDSNPs as verified by the markedly increased spin–orbit coupling (SOC) constants in the quantum mechanical calculation, as well as the enlarged ISC rate constants in the transient absorption spectra analysis. Moreover, two more energy-transfer pathways can be exploited to achieve additional PDT effect: (1) S_1_ state of MC540 transfers energy to ground state of Ce6 in at least 23 ps and (2) T_1_ state of MC540 offers energy to T_1_ state of Ce6 in the presence of CDSNPs. All above results induce a larger triplet-sate quantum yields *Φ*_*T*_ and subsequent highly efficient UC-PDT effect.

**Figure 1:**
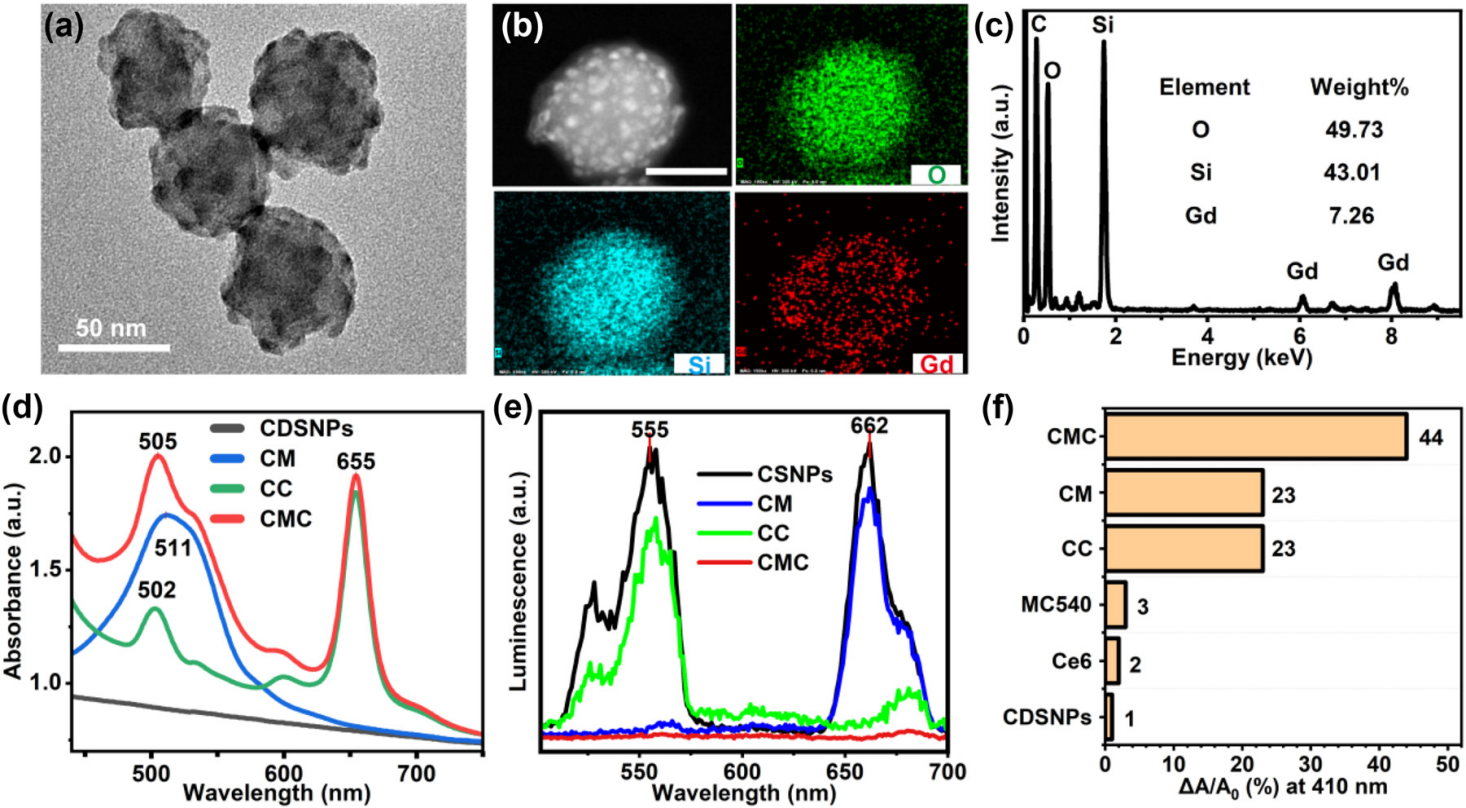
Characterization of structures and optical properties. (a) TEM micrograph of the CDSNPs. (b) HAADF-STEM image (scale bar: 50 nm), EDX elemental mapping images of O, Si, and Gd elements and corresponding EDX spectroscopy (c). (d) Absorption spectra of CDSNPs and PSs loaded CDSNPs. Here, CM, CC, and CMC denote CDSNPs@MC540, CDSNPs@Ce6, and CDSNPs@MC540/Ce6 in this Figure for simple annotation. (e) Upconversion luminescence spectra of CDSNPs, CM, CC, and CMC in PBS under the 980 nm excitation. (f) Absorption reduction of DPBF at 410 nm for assessing the photodynamic performance of CDSNPs, Ce6, MC540, CC, CM, and CMC. Here, CMC denotes CDSNPs@(^1^/_2_MC540 + ^1^/_2_Ce6).

The morphology of fabricated CDSNPs characterized by transmission electron microscope (TEM) indicates that some nanodots (around 5 nm) evenly distribute in nanospheres with a diameter approximately 50 nm (Figure 1(a)). The remarkable monodispersity, uniform size distribution and excellent photostability suggest their suitability for applications in aqueous biological environments (Figures S1–S2). High-angle annular dark-field scanning TEM (HAADF-STEM) images and energy-dispersive X-ray (EDX) mapping in Figure 1(b) illustrate the Gd elements predominantly distribute in outer layer while Si elements largely locate in the central core. EDX spectroscopy and line scanning curves in Figures 1(c) and S3 also exhibit the overall mass percentages of O, Si, and Gd elements.

Absorption spectra of CDSNPs and MC540/Ce6 loaded CDSNPs are presented in Figure 1(d). CDSNPs exhibit no obvious absorption peaks in visible light region but an increasing absorption intensity towards UV region. The prominent absorption of CDSNPs@MC540 is peaked around 511 nm corresponding to the characteristic absorption of MC540. The absorption curve of CDSNPs@Ce6 shows two distinct Q absorption bands including a strong absorption peak at 655 nm and a weaker absorption peak at 502 nm, attributed to Ce6 molecules. CDSNPs@MC540/Ce6 possesses the stronger and broader absorption peaks at both 505 nm and 655 nm due to the spectral integral sum of MC540 and Ce6 attached on CDSNPs.

Figure 1(e) displays the photoluminescence spectra of CDSNPs and MC540/Ce6 loaded CDSNPs in PBS under the excitation of 980 nm laser. With the suitable doping concentration of rare earth ions, marked red light centered at 662 nm and green light centered at 555 nm were observed for CDSNPs in aqueous solution (Figure S4). CDSNPs@MC540 displays robust luminescence only at 662 nm, while CDSNPs@Ce6 only remains strong green emission at 555 nm. Furthermore, almost no photoluminescence was detected by the spectrometer for CDSNPs@MC540/Ce6, indicating that the two emission bands of CDSNPs were completely absorbed by the two PSs owing to the FRET process.

To characterize the UC-PDT performance of the dual-PS system, we monitored changes in 1,3-diphenylisobenzofuran (DPBF) absorption at 410 nm when subjected to samples irradiated with a 980 nm laser for 10 min. This monitoring was conducted because the generation of singlet oxygen would lead to a reduction in DPBF absorption at 410 nm. Figure 1(f) demonstrates CDSNPs, MC540, or Ce6 alone hardly produce singlet oxygen under 980 nm laser irradiation, whereas CDSNPs loaded with either MC540 or Ce6 cause approximately 23 % reduction in DPBF absorption, suggesting efficient singlet oxygen generation. Since MC540 and Ce6 show negligible absorption at 980 nm (Figure S5), the excitation sources here should be produced *in situ* by the upconverted luminescence of CDSNPs. Notably, CDSNPs@(^1^/_2_MC540 + ^1^/_2_Ce6) induced a 43 % absorbance decrease of DBPF, significantly higher than the 23 % reduction caused by CDSNPs@single-PS, inferring a pronounced synergistic effect between MC540 and Ce6 on the surface of CDSNPs.

### 2.2 Excited-state characteristics

**Figure 2:**
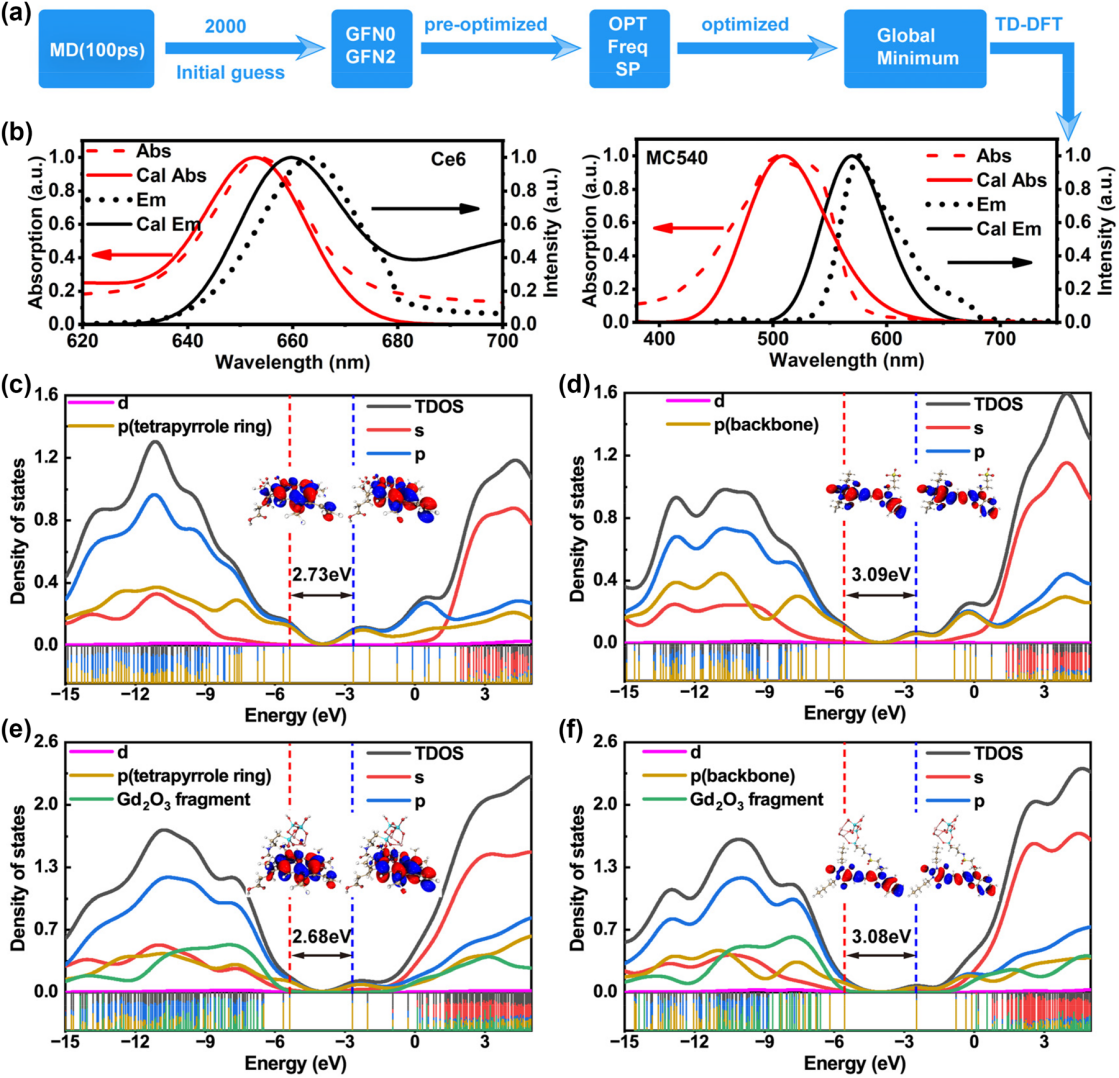
Geometry optimizations and electronic structures. (a) Conformational searching procedure for Ce6 and MC540 molecules. (b) The simulated (dotted lines and dashed lines) and experimental (solid lines) absorption and emission spectra for Ce6 and MC540 in PBS. (c, e) DOS of Ce6 and Gd/Ce6 (simplified CDSNPs@Ce6 model); (d, f) DOS of MC540 and Gd/MC540 (simplified CDSNPs@MC540 model). Vertical dash lines in red and blue mark out positions of HOMOs and LUMOs energy level. Distances between vertical red lines and vertical blue lines represent the HOMO-LUMO energy gaps. Insets on vertical dash lines in red and blue show isosurface maps of HOMOs and LUMOs.

To gain a better understanding of how the CDSNPs@dual-PS functioned as the highly efficient UC-PDT agents, we employed quantum mechanical calculations to investigate the electronic excitation behaviors of the first excited singlet states and the intersystem crossing between these first singlet states and triplet states of PSs in the presence of CDSNPs. Given the numerous rotatable chemical bonds in PSs, especially in MC540, we conducted a conformational search to identify the nearest global energy minima (Figure 2(a)). Initially, a set of structures (conformational ensembles) were generated using molecular dynamics simulations and pre-optimized with GFN0 and GFN2 methods. Subsequently, DFT methods were applied to optimize these structures and select the conformations with the lowest energy. The primary conformations of PSs in water were determined (Figure S6(a, b)), and TD-DFT methods were applied at CAM-B3LYP/def2-TZVP level to calculate the absorption and emission spectra. The calculated maximum absorption and emission wavelengths are 652.7 nm and 660.0 nm for Ce6 and 509.4 nm and 569.4 nm for MC540 (Figure 2(b)). These values align well with our experimental results, signifying that the chosen conformations and DFT/TD-DFT methods are appropriate for our study.

Based on the optimized structures of two PSs mentioned above, we proceeded to construct models for CDSNPs@Ce6 and CDSNPs@MC540 (i.e., Gd/Ce6 and Gd/MC540) using amide or sulfa linkers in accordance with the prepared nanocomposites (Figure S6(c, d)). The geometry optimizations and excited-state calculations were further performed using DFT and TD-DFT methods. The computational results reveal that the absorption peak at 654.0 nm for Ce6 or Gd/Ce6 is mainly contributed from the excitation configuration (over 83 %) between the highest occupied molecular orbital (HOMO) and the lowest unoccupied molecular orbital (LUMO). Similarly, the peak around 504.0 nm for MC540 or Gd/MC540 is predominantly attributed to the HOMO → LUMO excitation configuration (over 98 %). Both the HOMOs and LUMOs of Ce6 and MC540 have nodal planes on the tetrapyrrolic macrocyclic or backbone in the absence or presence of rare earth (Figure S7), demonstrating the characteristic of π MOs in conjugated organic systems. Hence, the HOMOs → LUMOs transitions are attributed to π → π* excitation. The HOMO-LUMO energy gaps for both Gd/Ce6 and Gd/MC540 are over 2.6 eV, slightly lower than those of free PSs. For more detailed energy-level distribution and orbital decomposition of MOs, it can be intuitively visualized by a density-of-state (DOS) diagram, where the value of the DOS curve reflects the number of MOs in the unit energy interval at the corresponding energy (Figure 2(c–f)). DOS curves indicate that the HOMOs and LUMOs in excitation primarily consist of p orbitals, specifically those in the tetrapyrrole ring of Ce6 and the backbone of MC540. The Gd_2_O_3_ fragments negligibly contribute to HOMOs and LUMOs but participate in those MOs whose energy is lower than HOMOs and higher than LUMOs (Figure 2(e and f)). It is concluded that the introduction of rare earth gadolinium retains the π → π* excitation characters and reduce the HOMO-LUMO gaps, which may facilitate the electronic excitation.

**Figure 3:**
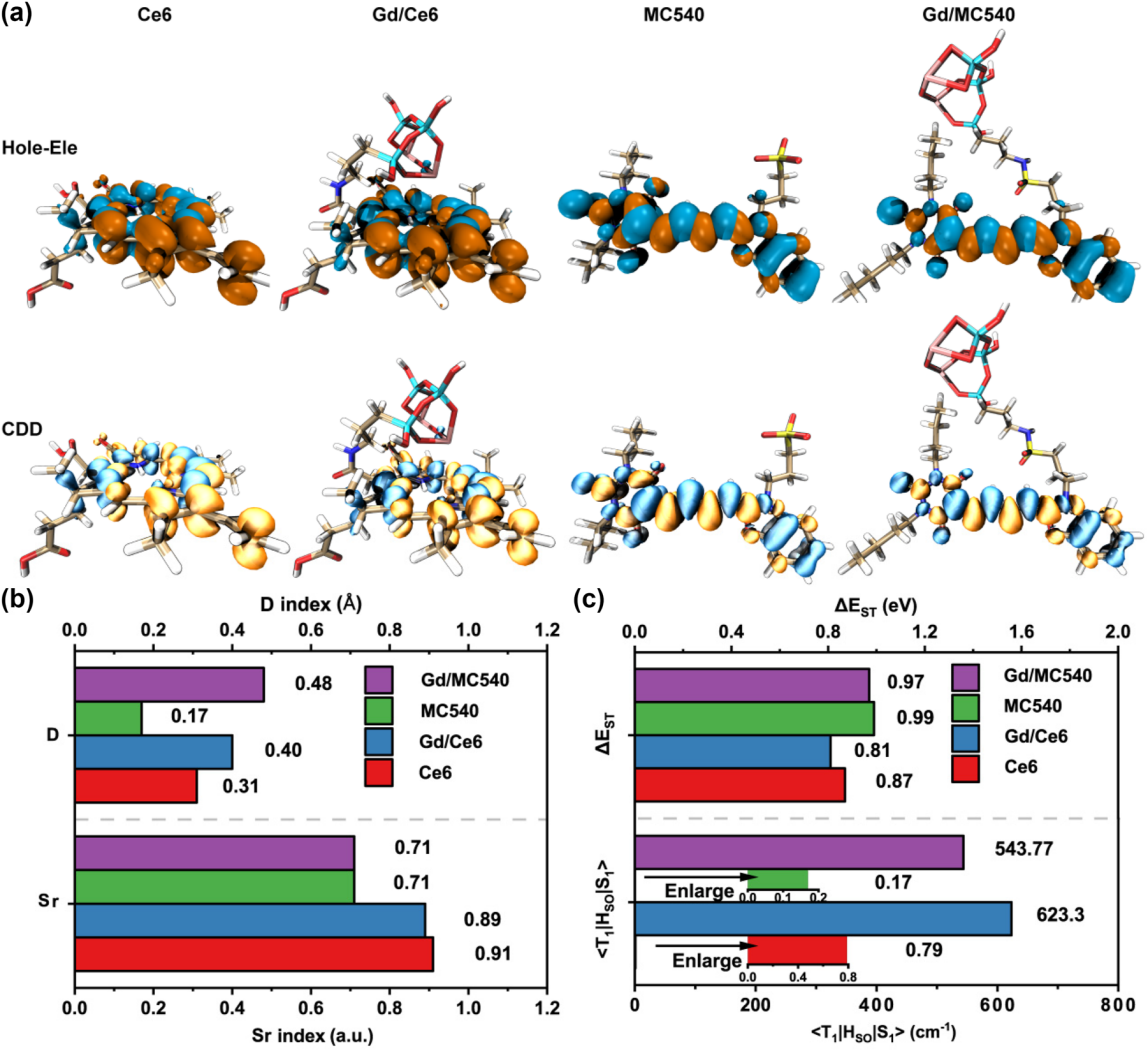
Electron excitation analysis and spin–orbit coupling. (a) The distribution analysis of hole (cyan) and electron (orange) and CDD maps. Yellow and blue regions in CDD maps denote the increase and decrease of electron density, respectively. (b) *D* and Sr index values. *D* index represents the centroid distance of hole and electron distribution. Sr index denotes the overlap content of hole and electron distribution. (c) The calculated energy gaps (Δ*E*_ST_) and spin–orbit coupling matrix elements (<T_1_|H_SO_|S_1_>) between the first singlet excited S_1_ states and the first triplet excited T_1_ states.

To characterize the electron-excitation natures of Ce6 and MC540 under the influence of rare earth, the hole-electron, and charge density difference (CDD) analyses for S_1_ states were performed using Multiwfn [21], [22] and visualized in Figure 3(a) with VMD software [23]. *D* and S_r_ indices were also calculated to illustrate the excitation nature in Figure 3(b). The hole (cyan) and electron (orange) distributions of Ce6 and Gd/Ce6 indicate the character of π → π* transition in accordance with the HOMO-LUMO mapping. The CDD maps reveal the localized excitation nature, since the increased charge density (yellow) and the decreased charge density (blue) locate on the tetrapyrrole ring alternately. The Sr index values of over 89 %, representing a significant overlap between hole and electron, confirm the localized excitation characteristic. Additionally, the calculated centroid distance (*D* index) between hole and electron for Gd/Ce6 is 0.4 Å, slightly larger than the 0.31 Å for Ce6. Regarding MC540 and Gd/MC540, the hole-electron distributions alternately distributing from benzoxazole group to thiobarbiturate terminal overlap well with HOMOs and LUMOs separately, indicating primary nature of π → π* transition. The Localized excitation characters were also confirmed by the CDD distributions and Sr index values of 71 %. Similarly, *D* index increases from 0.17 Å to 0.48 Å. Furthermore, the transition density matrix contributed by specific atoms was also calculated to analyze the hole-electron localization and the probability for the S_0_ → S_1_ transition (Figure S8). It seems electrons tend to transfer towards moiety close to rare earth Gd, in both Ce6 and MC540 when coupled with the rare earth Gd fragment.

Photodynamic performance is directly related to ISC process between excited singlet states and triplet states, in addition to electronic excitations of singlet states [24]. We calculated spin–orbit coupling matrix element (SOCME, <T_1_|H_SO_|S_1_>) and energy gap between the first excited singlet state S_1_ and triplet state T_1_ (Δ*E*_ST_) in Figure 3(c). The energy gap Δ*E*_ST_ of Gd/Ce6 falls from 0.87 eV to 0.81 eV, while the SOCME experiences a substantial increase from 0.79 cm^−1^ to 623.3 cm^−1^, representing a remarkable 788 % increment, compared to Ce6. Regarding Gd/MC540, the energy gap Δ*E*_ST_ decreases from 0.99 eV to 0.97 eV and the SOCME value increases from 0.17 cm^−1^ to 543.77 cm^−1^ with 3197 % amplification, compared to MC540. Generally, according to spin selection rule, transitions from the singlet S_1_ state to the triplet T_1_ state are spin-forbidden, resulting in low SOCME values. When heavy atoms are present in the system, SOC effect is significantly enhanced, inducing considerable SOCME values [25]. In our study, the introduction of rare earth element Gd greatly facilitates ISC from S_1_ to T_1_ through a strong SOC interaction. Both the significant SOCME value and the reduced Δ*E*_ST_ promote the ISC process, leading to increased singlet oxygen generation.

### 2.3 Transient photoexcitation dynamics

**Figure 4:**
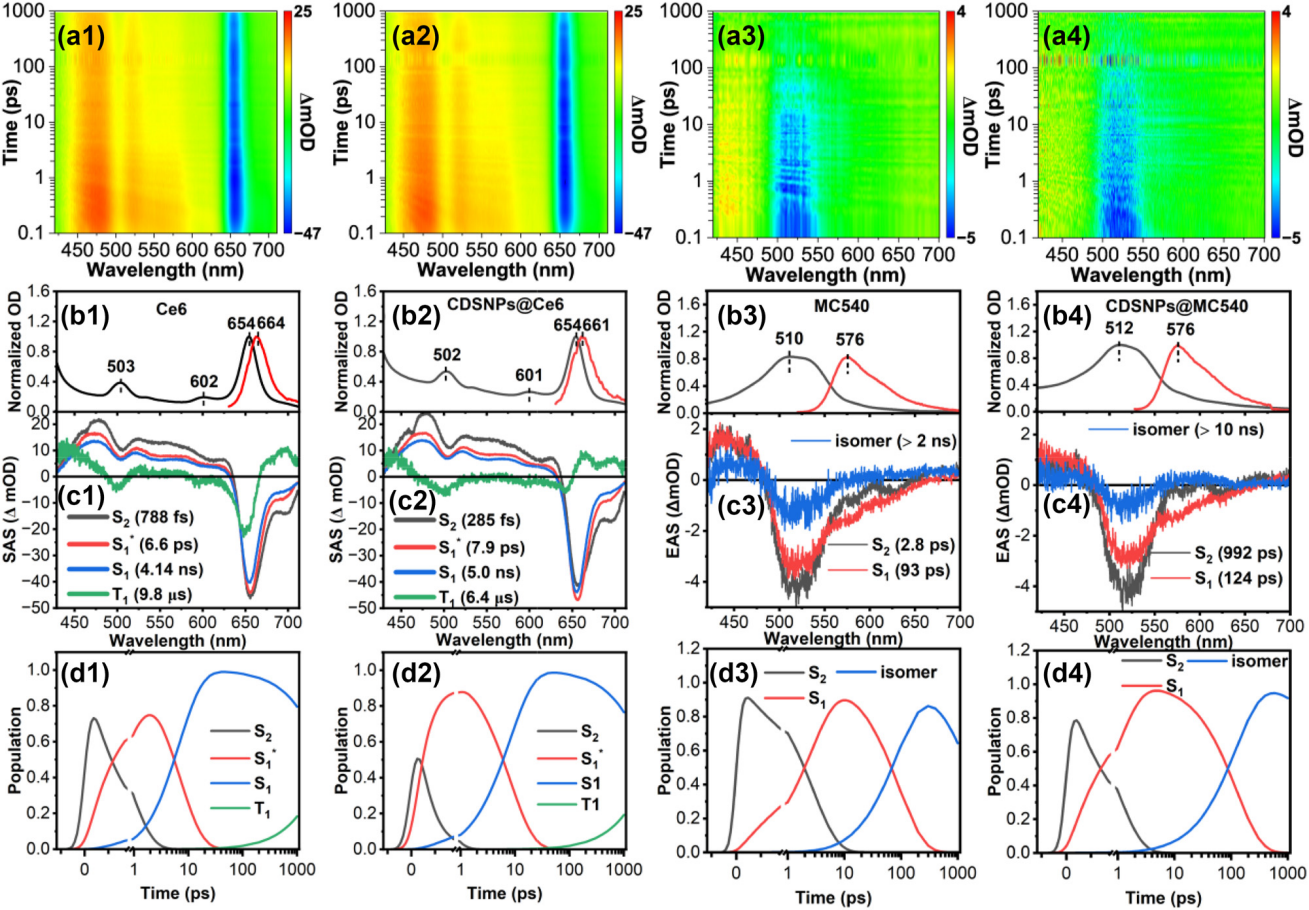
The femtosecond to nanosecond transient absorption spectroscopy. (a1–a4) Transient absorption contour maps. (b1–b4) Ground-state absorption (black) and fluorescence spectra (red). (c1–c4) Species or evolution associated spectra. (d1–d4) Population evolution of corresponding species. Pump wavelength is 400 nm.

To investigate the impact of rare earth elements on the excited states of MC540 and Ce6 experimentally, time-resolved TA spectra spanning from femtosecond to nanosecond timescales were acquired in Figures 4 and S9. Microsecond TA spectra were additionally recorded in Figures S10–S11 under both deaerated and aerated conditions in PBS to determine the triplet properties. The detailed methods are described in Supplementary Materials and the estimated triplet parameters are listed in Table 1. The TA spectra of Ce6 and CDSNPs@Ce6 within femtosecond to nanosecond exhibit pronounced excited-state absorption bands at 427–635 nm, with a peak at 470 nm, along with ground-state bleaching bands from 635 nm to 711 nm, reaching a minimum at 654 nm (Figure 4(a1–c1, a2–c2)), respectively. Besides, their TA spectra in the microsecond time domain reveal that excited-state absorption bands are centered around 440 nm in both deaerated and aerated PBS, bearing a close resemblance to the triplet absorption of ZnCe6 [26]. A favorable fit to the kinetics at 440 nm indicates the triplet lifetime of Ce6 is approximately 157 μs in deaerated PBS and 9.79 μs in aerated PBS. Comparatively, CDSNPs@Ce6 demonstrates a longer triplet lifetime in deaerated PBS and a shorter one in aerated PBS, signifying the larger triplet-sate quenching rate and proportion caused by oxygen. The triplet quantum yield *Φ*_*T*_ and the ISC rate *k*_isc_ are about 0.42 and 1.02 × 10^8^ s^−1^ for Ce6, while CDSNPs@Ce6 displays larger *Φ*_*T*_ of 0.62 and *k*_isc_ of 1.24 × 10^8^ s^−1^, confirming the promotion effect of rare earth nanoparticle on ISC from S_1_ states to T_1_ states of PSs. The TA spectra of MC540 and CDSNPs@MC540 from femtosecond to nanosecond are predominantly constituted by excited-state absorption at 420–480 nm and ground-state bleaching or stimulated emission around 480–660 nm (Figure 4(a3–c3, a4–c4)). The excited-state absorption maximum of the CDSNPs@MC540 is situated at approximately 430 nm, exhibiting a blueshift about 10 nm compared to MC540. The TA spectra of MC540/Ce6 resemble those of Ce6 across the entire time domain, potentially arising from the Soret-band excitation. Nonetheless, the decay kinetics at 440 nm for MC540/Ce6 in deaerated PBS exhibits not only a short lifetime of 238 μs but a long lifetime of 731 μs, while MC540/Ce6 in aerated PBS displays only one short lifetime of about 6.25 μs. According to literature [27], the 731 μs should be attributed to the triplet state of MC540 and the 238 μs should be ascribed to Ce6, even if slightly longer than triplet lifetime of Ce6 in deaerated PBS. Besides, the microsecond TA spectrum of MC540/Ce6 in deaerated PBS reveals a remarkable absorption band at 500–620 nm compared to the spectra of Ce6 and CDSNPs@Ce6. The 540 nm kinetic exhibits a short lifetime of about 216 μs with a negative amplitude of −0.001 and an ultra-long lifetime of 7232 μs with a positive amplitude of 0.0068 while Ce6 or CDSNPs@Ce6 only possesses one short lifetime of less than 200 μs. The short lifetime arises from the triplet state of Ce6 while the ultra-long one of 7232 μs should be contributed from photoisomer of MC540 [27]. The presence of both negative and positive amplitudes signifies the energy-transfer from the photoisomer of MC540 to the triplet state of Ce6. This complexing endows Ce6 a larger triplet quantum yield of 0.50, an accelerated ISC rate of 1.18 × 10^8^ s^−1^ and an elevated oxygen quenching rate of 0.577 × 10^9^ M^−1^ s^−1^ compared to isolated Ce6, further reinforcing the synergistic effect between two PSs.

**Table 1:** Summary of triplet state parameters for Ce6, MC540/Ce6 and CDSNPs@Ce6 in PBS.

Compound	$\tau_{T}^{0}$ ^a^ (μs)	*τ*_*T*_^b^ (μs)	$k_{q}^{T}$ ^c^ (10^9^ M^−1^ s^−1^)	$P_{O_{2}}^{T}$ ^d^	*ε*_*T*_^e^ (M^−1^ cm^−1^)	*k*_*isc*_^f^ (10^8^ s^−1^)	Φ_*T*_^g^
Ce6	157 ± 0.8	9.79 ± 0.05	0.355	0.938	30,764	1.02	0.42
MC540/Ce6	238 ± 6	6.25 ± 0.04	0.577	0.974	32,025	1.18	0.50
CDSNPs/Ce6	168.3 ± 0.2	6.39 ± 0.03	0.558	0.963	32,179	1.24	0.62

^a^The triplet lifetimes in deaerated PBS. ^b^The triplet lifetimes in aerated PBS. ^c^The oxygen quenching rate constants of triplet state. ^d^The proportion of triplet states quenched by O_2_. ^e^The triplet state molar extinction coefficients at the maximum triplet absorption. ^f^The intersystem-crossing rate constants. ^g^The triplet quantum yields were obtained using comparative actinometry method.

**Scheme 2:**
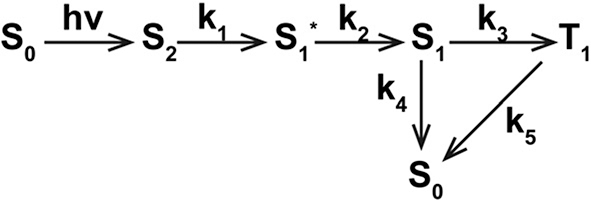
Kinetic model used for target analysis of transient absorption data of samples containing Ce6 in PBS upon 400 nm excitation.

**Scheme 3:**
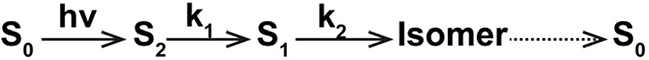
Kinetic model used for sequential analysis of transient absorption data of MC540/CDSNPs@MC540 in PBS upon 400 nm excitation.

To perform a more comprehensive analysis of the obtained TA spectra, we employed a global fit analysis based on singular value decomposition. Since detailed triplet properties as well as S_1_ decay kinetics have been obtained for samples containing Ce6 (Table 1 and Figure S12), target model was applied to extract the species-associated spectra (SAS) for these samples in Scheme 2. However, due to the complexity of S_1_ state such as photoisomerization, sequential model was utilized to derive the evolution-associated spectra (EAS) for MC540 and CDSNPs@MC540 in Scheme 3. In the target model, electrons from S_1_ either return to ground state S_0_ by emitting fluorescence or proceed to the first excited triplet state T_1_ through an ISC process, ignoring other non-radiative pathways. Subsequently, triplet state T_1_ decays to the ground state at the rate of *k*_5_. Both *k*_isc_ (*k*_3_) and *k*_5_ (1/*τ*_*T*_) have been determined in Table 1. *k*_4_ can be derived by subtracting *k*_isc_ from 1/*τ*_fl_. Therefore, *k*_3_, *k*_4_ and *k*_5_ were fixed in the target analysis for the precise examination of *k*_1_ and *k*_2_. We obtained four SAS with five distinct time constants for scenarios involving Ce6, depicted in Figures 4(c1, c2) and S9(c1, c2). The corresponding population evolution is illustrated in Figures 4(d1, d2) and S9(d1, d2). The quality of the fits is depicted in Figures S14–S15 and S18–S19. Considering the Soret-band excitation, the first species for Ce6-based samples should be assigned to S_2_ state. The S_2_ state decays to a vibrationally “hot” S_1_ state (S_1_*) through an internal conversion process with the rate of *k*_1_. Consequently, the lifetime of S_2_ was determined to be 788, 285, 1000, and 333 fs for Ce6, CDSNPs@Ce6, MC540/Ce6, and CDSNPs@MC540/Ce6, respectively. Evidently, the presence of rare earth nanoparticles significantly accelerates the internal conversion process. As the energy gaps between S_2_ and S_1_ (Δ*E*(S_2_–S_1_)) are evaluated to be comparable for them according to variations in the steady-state absorption of Soret-band and Q-band (Figures 4 and S9), the energy gap law is not applicable in our study [28]. It is inclined to the explanation that the introduction of CDSNPs results in ruffling of the macrocycle and the increased non-planar structures accelerate the rate of radiationless decay [29]. The S_1_* typically undergoes decay to the lowest vibrational state (S_1_) through a vibrational cooling process with the time from one to several tens of picoseconds [30]. In this study, the time constant (1/*k*_2_) was determined as 6.6, 7.9, 29.3, and 86 ps for Ce6, CDSNPs@Ce6, MC540/Ce6, and CDSNPs@MC540/Ce6, respectively. It suggests that the vibrational cooling time increases with the raised structural size, which should be attributed to larger molecular systems potentially requiring more time to transfer excess vibrational energy to surrounding solvents. The fitted SAS of T_1_ (green line) are blue shifted approximately 30 nm for the maximum peaks compared to SAS of S_1_, closely resembling those TA spectra in microsecond time domain (Figures S10–S11). This alignment further substantiates the rationality of our proposed target model.

In Figure 4(c3, c4), three EAS with three different time constants were obtained for MC540 and CDSNPs@MC540 using the sequential model in Scheme 3. The corresponding population evolution and the goodness of the fits are presented in Figures 4(d3, d4) and S16–S17, respectively. According to our quantum mechanical calculations, the S_2_ state of MC540 is situated at 427.33 nm; therefore, the 400 nm pump instantaneously excites MC540 to the S_2_ state, as shown in black spectra of Figure 4(c3, c4). Subsequently, the spectra of S_2_ state evolve to the red EAS in 2.8 ps for MC540 and 992 fs for CDSNPs@MC540. The new EAS broaden due to the presence of stimulated emission in contrast to the EAS of S_2_. Therefore, we assigned this species as S_1_ state. Analogous to cases involving Ce6, the internal conversion process of MC540 is expedited due to the influence of CDSNPs. The EAS of S_1_ evolves to subsequent blue EAS in 93 ps for MC540 and 124 ps for CDSNPs@MC540. These two time constants are marginally shorter than fluorescence lifetimes measured with a streak camera (Figure S13). Additionally, blue EAS emerge positive bands at 570–625 nm except for the recovery of ground-state bleaching bands. These positive bands closely resemble the TA of photoisomers of MC540 in methanol solution [31]. Moreover, owing to the higher yield of photoisomers in comparison to the triplet state and fluorescence, S_1_ state of MC540 predominantly decays into photoisomers under laser irradiation. Consequently, this component has been attributed to isomer of MC540. Eventually, these isomers slowly decay to the ground state.

### 2.4 Time-resolved fluorescence spectroscopy

**Figure 5:**
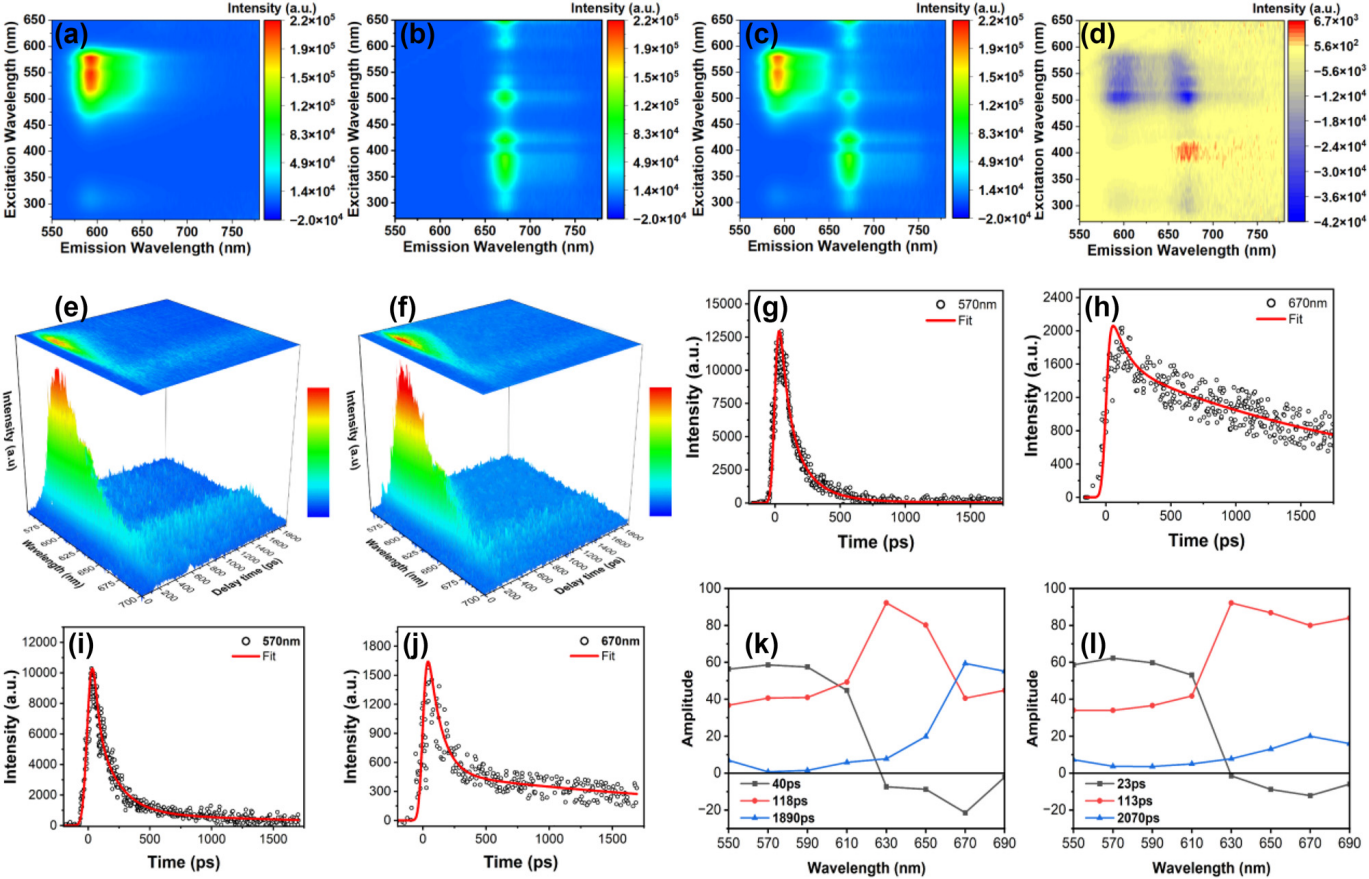
The energy-transfer revealed by both steady-state and time-resolved fluorescence spectroscopies. (a–c) Photoluminescence contour maps of MC540, Ce6 and MC540/Ce6. (d) Difference spectra plotted with (c) subtracting (a) and (b). (e, f) Time-resolved fluorescence spectra of MC540/Ce6 and CDSNPs@MC540/Ce6 under the excitation of 500 nm laser (*λ*_ex_ = 500 nm) at room temperature. The color bar reflects the relative intensity of spectra. The characteristic decay curves (black circles) and fits (red lines) at 570 nm and 670 nm for (g, h) MC540/Ce6 and (i, j) CDSNPs@MC540/Ce6. (k, l) The fluorescence decay-associated spectra of MC540/Ce6 and CDSNPs@MC540/Ce6, *λ*_ex_ = 500 nm.

The improved photodynamic performance of CDSNPs@(^1^/_2_MC540 + ^1^/_2_Ce6) in the singlet oxygen detection experiment can be partly ascribed to the energy transfer between MC540 and Ce6. This is due to the overlap between the emission spectrum of MC540 and the absorption spectrum of Ce6 (Figure S20). Hence, steady-state and time-resolved fluorescence spectroscopies were utilized to elucidate the specific energy transfer pathways and rates between MC540 and Ce6. The absorption spectrum of MC540/Ce6 in DMSO is characterized by an increased absorbance for Ce6 and a decreased absorbance for MC540 (Figure S21). Figure 5(a–c) depicts photoluminescence contour maps recorded under the excitation of 275–650 nm. Figure 5(d) illustrates net variation in photoluminescence for MC540/Ce6 after subtracting contributions of isolated MC540 and Ce6. Importantly, compositing of MC540 and Ce6 results in an overall reduction in photoluminescence intensity for MC540, but there is still an increase in emission for Ce6, particularly when excited at 400 nm. The alterations in emission intensity between MC540 and Ce6 further confirm the existence of energy transfer.

Three-dimensional (3D) time-resolved fluorescence spectra of MC540/Ce6 and CDSNPs@MC540/Ce6 are presented in Figure 5(e and f), respectively. The excitation wavelength was set to 500 nm to primarily excite MC540 molecules because it closely matches the maximum absorption position of MC540. A fluorescence peak at about 576 nm was observed which represents the emission of MC540. With the time delays, the emission band of MC540 around 576 nm decays along the rise of the Ce6 emission at 664 nm, suggesting the energy transferred from MC540 to Ce6. In the presence of CDSNPs, the spectral evolution maintains consistent characteristics, with the exception of accelerated decay and rise rates. To ascertain the energy-transfer rates from MC540 to Ce6, a deconvolution procedure based on global optimization algorithm was applied. Three-exponential decay model and Monte-Carlo method were adopted in the deconvolution process to obtain the best fitting curves (Figures 5(g–j) and S22–S23). The deconvolution results including three decay time constants of 40, 118, and 1890 ps for MC540/Ce6, three decay constants of 23, 113, and 2070 ps for CDSNPs@MC540/Ce6 and corresponding amplitudes are documented in Table 2.

**Table 2:** The deconvolution results for MC540/Ce6 and CDSNPs@MC540/Ce6 (*λ*_ex_ = 500 nm).

	550 nm	570 nm	590 nm	610 nm	630 nm	650 nm	670 nm	690 nm
**MC540/Ce6**								
40 ps	56.32	58.63	57.52	44.78	−7.33	−8.73	−21.45	−2.15
118 ps	36.75	40.65	41.01	49.36	92.19	80.17	40.59	44.81
1890 ps	6.93	0.72	1.47	5.86	7.81	19.83	59.41	55.19
**CDSNPs@MC540/Ce6**								
23 ps	58.65	62.28	59.77	53.12	−1.41	−8.78	−12.14	−5.93
113 ps	34.08	34	36.63	41.82	92.17	86.87	80	84.03
2070 ps	7.28	3.72	3.61	5.06	7.83	13.13	20	15.97

Additionally, fluorescence decay-associated spectra (FDAS), derived from the deconvolution results, are presented in Figure 5(k, l). Time constants of 40 ps for MC540/Ce6 and 23 ps for CDSNPs@MC540/Ce6 were assigned as the time required for energy-transfer from MC540 to Ce6. This assignment is based on the observation of positive amplitudes in the blue region peaking at approximately 570 nm and negative amplitudes in the red region around 670 nm. Positive amplitudes signify the fluorescence decay process, while negative amplitudes indicate the fluorescence rise stage, providing evidence of energy-transfer from a donor to an acceptor. The shorter time constant of 23 ps for CDSNPs@MC540/Ce6 in comparison to the 40 ps for MC540/Ce6 implies a higher rate of energy-transfer, probably attributed to the closer proximity of two PSs on the surface of CDSNPs. Time constants of 118 ps for MC540/Ce6 and 113 ps for CDSNPs@MC540/Ce6 correspond to the fluorescence lifetime of MC540 [27]. Furthermore, time constants of 1890 ps for MC540/Ce6 and 2070 ps for CDSNPs@MC540/Ce6 can be ascribed to the emission from Ce6 because positive amplitudes in the FDAS exhibit a maximum peak around 670 nm. The time-resolved fluorescence results indicate the existence of an additional FRET pathway from MC540 to Ce6, contributing to the enhancement of the synergistic PDT effect.

### 2.5 PDT performance

**Figure 6:**
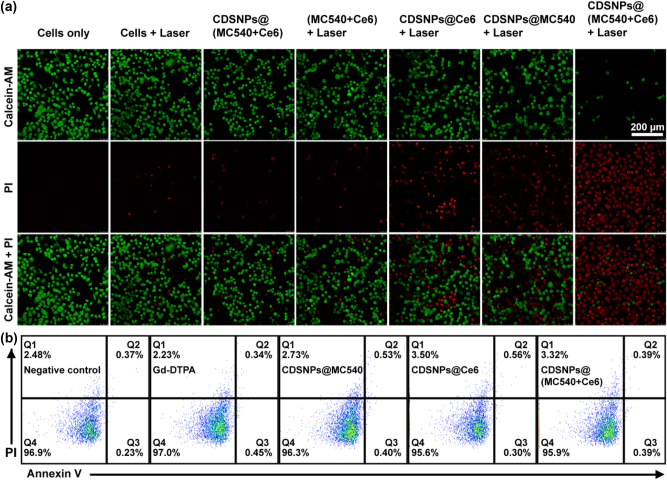
UC-PDT effect and dark toxicity of dual-PS system *in vitro*. (a) Live/dead staining images of CNE-2 cells after the treatment with different samples and a 980 nm laser irradiation at the power densities of 0.7 W cm^−2^. Scale bar at the right upper corner is 200 µm for all images. (b) Apoptosis analysis of L-O2 cells stained with Annexin-V/PI after incubated with PBS, commercial Gd-DTPA, CDSNPs@MC540, CDSNPs@Ce6 and CDSNPs@(MC540+Ce6) for 48 h. Q1 is the region where cells should not turn up; Q2 and Q3 represent the apoptosis cells in the later and early stages, respectively; and Q4 displays the normal living cells.

To further evaluate the UC-PDT effect of samples on biological system, ROS generation in CNE-2 nasopharyngeal cancer cells was first measured by monitoring the fluorescence intensity of the ROS sensor using the flow cytometry techniques before the PDT experiment. After treated with folate-PEGylated PSs-loaded CDSNPs and the irradiation of 980 nm laser, much higher fluorescence intensity was observed in cancer cells compared to the control groups, demonstrating an improved ROS generation (Figure S24). Furthermore, live/dead assay was performed after the CNE-2 cells were incubated with folate-PEGylated PSs-conjugated CDSNPs and exposed to a 980 nm NIR laser for 10 min (Figure 6(a)). Cell viability was also calculated based on the quantity percentage of live cells using Image J software (Figure S25). Propidium iodide (PI) was used to stain the nuclei of dead cells because it cannot cross the cell membrane of live cells and emit strong red fluorescence until membrane is compromised. Calcein-AM was employed to stain live cells since it functioned as a green fluorescent dye after converted to calcein by intracellular esterases of viable cells. According to live/dead assay, group treated upon NIR laser irradiation caused over 31 % mortality of cells. However, group treated with identical dose of CDSNPs@(MC540+Ce6) and NIR laser irradiation was almost all stained with red fluorescence and the proportion of dead cells was up to 92 %, which demonstrates the significantly enhanced PDT effect on tumor cells. Besides, the dark toxicity of nanocomposites to normal human liver L-O2 cells stained with Annexin-V/PI under the strict protection from light was also evaluated using the apoptosis analysis performed on a flow cytometry (Figures 6(b) and S26). The apoptosis analysis exhibits the dual-PS coupled CDSNPs caused less than 1 % total apoptosis, which reveals nanocomposites have a negligible effect on cell survival and possess a satisfactory biocompatibility in comparison to the negative groups and clinical commercial Gd-DTPA. The bioassay results demonstrate that the dual-PS nanosystem itself has no cytotoxicity but enables an excellent anti-tumor performance under NIR laser irradiation, making it promising in clinical cancer treatment.

## 3 Conclusions

In conclusion, we have prepared unique SiO_2_@Gd_2_O_3_:Yb^3+^/Er^3+^/Li^+^ core@dotted-shell nanoparticles (CDSNPs) and covalently linked MC540 and Ce6 PSs to CDSNPs, thereby establishing a dual-PS UC-PDT system. The CDSNPs exhibit exceptional upconversion red and green luminescence efficiency. The dual-color emission spectra of CDSNPs are substantially overlapped with absorption spectra of two PSs, leading to a remarkable FRET efficiency. The efficient energy transfer enables the efficient activation of each PS for photodynamic effects. Quantum mechanical calculations elucidate the role of rare earth element in promoting charge transfer of PSs without altering their localized excitation characteristics. And a substantial enhancement of the SOC of rare-earth-coupled PS facilitates ISC process, consequently increasing the population of triplet states. Transient absorption spectroscopy reveals that the rare-earth CDSNPs markedly expedite the internal conversion and ISC process of PSs, leading to an enhanced quantum yield of triplet state. Furthermore, the coupling of the dual PSs alone also increases the triplet quantum yield and ISC rate constant of Ce6, indicating the enhancement effect of MC540 on the photodynamic capabilities of Ce6. Time-resolved fluorescence spectroscopy unveils an energy transfer process from MC540 to Ce6, with the energy transfer time reduced from approximately 40 ps–23 ps in the presence of rare-earth CDSNPs. This additional pathway of energy transfer further heightens the photodynamic effect of the dual PSs. *In vitro* experiments confirm the biocompatibility of the CDSNPs-based dual-PS system and its ability to induce a synergistically high-efficiency PDT effect on cancer cells under NIR laser irradiation. These results underscore the efficacy of the dual-PS photodynamic system developed in this study. And the elucidated mechanism can provide valuable insights for designing other efficient UC-PDT systems and facilitating their implementation in preclinical settings.

## 4 Methods

Experimental details are given in the Supplementary Material.

## Supplementary Material

Supplementary Material Details
