# Supplementary material for: Highly efficient upconversion photodynamic performance of rare-earth-coupled dual-photosensitizers: ultrafast experiments and excited-state calculations
Source: Nanophotonics. 2024 Feb 5;13(4):443–55. doi: 10.1515/nanoph-2023-0772 (PMC11501619; doi:10.1515/nanoph-2023-0772)
Supplement: Supplementary file 1 — Supplementary Material Details [file j_nanoph-2023-0772_suppl_001.pdf]

Supplementary Information

Highly efficient upconversion photodynamic performance of rare-earth-coupled dual-photosensitizers: Ultrafast experiments and excited-state calculations

Yubiao Yang,[1] Lei Zhang,[1] Chao Xiao,[1] Zhencheng Huang,[1] Fuli Zhao,[1]* and Jinchang Yin[2]*

[1]School of Physics, State Key Laboratory of Optoelectronic Materials and Technologies, Sun Yat-sen University, Guangzhou 510275, China

[2]School of Biomedical Engineering, Health Science Center, Shenzhen University Shenzhen 518060, China

*stszfl@mail.sysu.edu.cn and yinjch6@szu.edu.cn

Table of contents

1. Methods 3

1.1 Fabrication of SiO_2@Gd_2O_3:Yb^{3+}/Er^{3+}/Li^+ nanoparticles 3

1.2 Conjugation of CDSNPs with dual photosensitizers and targeting molecules 3

1.3 Characterization and optical measurements 3

1.4 Computational Methods 4

1.5 Transient absorption measurement 5

1.6 The methods to calculate the lifetimes and quantum yields of triplet state, ISC rate constants, etc. 6

1.7 Time-resolved fluorescence measurement 7

1.8 ROS generation and live/dead assay of CNE-2 cells for UC-PDT effects of CDSNPs@MC540/Ce6 8

1.9 Dark cytotoxicity assays 8

2. Supplementary figures 9

3. Supplementary tables 28

4. References 31

1. Methods

1.1 Fabrication of $SiO_2@Gd_2O_3:Yb^{3+}/Er^{3+}/Li^+$ nanoparticles

First, we synthesized monodisperse silica nanoparticles following previous reported approach with minor modifications [1]. Briefly, 5 mL of deionized water, 100 mL of ethanol and 10 mL of aqueous ammonia (25%) were added into a conical flask and mixed evenly in an electrothermostatic water bath at 40 °C. Then 3 ml of tetraethyl ortho-silicate (TEOS) was slowly added into the mixtures and followed gently stirring for 24 h. The synthesized silica nanoparticles were gained after centrifuged at 8500 rpm for 10 min and washed with deionized water and ethanol alternately 3 times. Second, the silica nanoparticles were re-dispersed into 100 ml of deionized water in a conical flask and added with 2.0 g of urea. Then 0.1 mmol of $Gd(NO_3)_3·6H_2O$, $Yb(NO_3)_3·5H_2O$, $Er(NO_3)_3·5H_2O$ and $LiNO_3·H_2O$ with the metal ions molar ratio of $Gd^{3+}:Yb^{3+}:Er^{3+}:Li^+$ = 86:5:2:7 was supplemented, and the mixtures were stirred vigorously for 5 h in a water bath at 80 °C. Then, the sesquioxide carbonate hydrate was deposited onto SiO_2 nanoparticle to form a thin nanoshell. After centrifuged at 8000 rpm for 8 min and washed with ethanol and deionized water alternately 2 times, precursors were collected, frozen in a refrigerator and then dried in a vacuum refrigerant drier at minus 40 °C for 48 h. The dried powders were calcined at 800 °C in an electric furnace for 5 h and cooled naturally, obtaining final $SiO_2@Gd_2O_3:Yb^{3+}/Er^{3+}/Li^+$ core@dotted-shell nanoparticles (CDSNPs).

1.2 Conjugation of CDSNPs with dual photosensitizers and targeting molecules

The prepared CDSNPs powders were re-dispersed ultrasonically in 50 mL of ethanol. Then, 0.3 mL of 3-Aminopropyltriethoxysilane (APTES) was supplemented and the mixtures were stirred for 12 h under reflux at 60 °C to obtain aminated CDSNPs. The aminated CDSNPs were washed with ethanol to remove residual reagents and dried in vacuum. Then aminated CDSNPs powders were re-dispersed in 20 mL of dimethyl sulfoxide (DMSO) ultrasonically. 2 mg of folate-poly(ethylene glycol)-carboxylic acid (folate-PEG-COOH), 2 mg of Ce6, 1 mg of MC540, 2 mg of 1-ethyl-3-(3-dimethylaminopropyl) carbodiimide (EDC) and 4 mg of N-hydroxysuccinimide (NHS) were supplemented into the dispersions followed stirring for 24 h. Finally, folate-PEGylated CDSNPs@Ce6/MC540 were obtained after purified ultrasonically with DMSO, ethanol, and deionized water in triplicate.

1.3 Characterization and optical measurements

The morphology and energy dispersive X-ray (EDX) spectra of CDSNPs was characterized on transmission electron microscope (TEM, FEI Tecnai G2 Spirit) equipped with a field emission gun operating at 120 kV. High-angle annular dark-field scanning TEM (HAADF-STEM) images, EDX elemental mapping and line scanning were recorded on a Tecnai G2 F30 instrument at an accelerating voltage of 300 kV. Absorption spectra of samples dispersed in 10 mm quartz cuvettes were acquired on a UV-Vis-NIR spectrophotometer (UV-3600). Upconversion photoluminescence spectra of CDSNPs and CDSNPs@Ce6/MC540 dispersed in phosphate buffered saline (PBS)

were recorded using an Edinburgh spectrofluorophotometer (FLS980) equipped with a 980 nm diode laser as the excitation source and R928P photomultiplier as the detector. The spot size and power of 980 nm laser were 5 mm × 8 mm and 0.63 W, respectively. Excitation and emission spectra of photosensitizers Ce6 and MC540 were recorded with excitation source of xenon lamp. In particular, two-dimensional (2D) emission spectra of Ce6 and MC540 from 550-780 nm were recorded with a long pass filter of 420 nm, an excitation slit of 0.8 nm and an emission slit of 1 nm at excitation wavelength between 270-650 nm with a stepping of 10 nm. Singlet oxygen ($^1O_2$) generation was detected to evaluate the PDT efficiency using 1,3-diphenylisobenzofuran (DPBF) probe method [2]. In our case, the decreased absorbance of DPBF solution at 410 nm in the presence of the same concentrations of Ce6, MC540, CDSNPs, CDSNPs@Ce6, CDSNPs@MC540 and CDSNPs@($^1/_2$MC540+$^1/_2$Ce6) were calculated separately representing corresponding production of $^1O_2$ under the irradiation of 980 nm laser for 10 min.

1.4 Computational methods

Conformational searching was finished to find the global energy minima of Ce6 and MC540 by a reasonable set of methods. First, molecular dynamics simulations with a simulation time of 100 ps and a simulation temperature of 400 K were achieved to find 2000 initial guess using xtb, a semi-empirical program package [3]. Second, GFN0-xTB and GFN2-xTB methods were adopted to perform sequential batch optimization of the obtained initial guesses to remove conformations with relatively high energy [4]. Third, remaining structures were optimized with DFT calculations at B3LYP/6-31G* level with Grimme's D3BJ dispersion correction (GD3BJ) to get the thermal correction to Gibbs free energy [5]. Solvent effects of water were considered by the universal solvation model based on solute electron density (SMD) [6]. Frequency calculations were also achieved at the same functional and basis set to obtain the reliable structures with no negative frequency. Furthermore, single-point calculations of the optimized structures were performed using double-hybrid functional and the quadruple-ζ def2-QZVPP basis set to improve the calculation accuracy, by ORCA program [7-9]. The implicit solvent model based on SMD was also employed to present the solvent environment. Finally, geometry conformations were ranked by Gibbs free energy calculated by the sum of single point energy with thermal correction to Gibbs free energy. Main conformations at room temperature were confirmed with Boltzmann distribution calculations using the Molclus software [10].

The absorption spectra of Ce6 and MC540 were calculated at the CAM-B3LYP/def2-TZVP level [8, 11] with GD3BJ corrections and SMD model of water. The emission spectra of Ce6 and MC540 were calculated on the optimized S_1 structure at the CAM-B3LYP/def2-TZVP level with GD3BJ and SMD of water. In comparison to experiment data, these spectra for Ce6 and MC540 were broadened using Gaussian function with a full width at half maximum of 240 cm^{-1} and 700 cm^{-1}, respectively.

Based on the optimal structures of Ce6 and MC540, simplified structures of

$SiO_2@Gd_2O_3$/Ce6 (abbreviated as Gd/Ce6) and $SiO_2@Gd_2O_3$/MC540 (abbreviated as Gd/MC540) were modeled according to the prepared nanocomposite. The structures involving PSs and rare earth coupled PSs were first optimized using the PBE0 exchange−correlation functional [12]. We used quasi-relativistic effective core potentials including 53 electrons in the core for Gd (ECP53MWB), together with their associated (7s6p5d)/[5s4p3d] (Gd) basis sets [13]. Def2-TZVP basis set was used for all other atoms (C, H, O, N, Si and S). The optimized geometries corresponding to local energy minima were confirmed by the absence of imaginary frequencies at the same level.

10 low-lying singlet excited states and 10 low-lying triplet excited states were calculated based on optimized geometries in the ground states for Gd/Ce6 and Gd/MC540 via TD-DFT at PBE0/def2-TZVP level. Multiwfn program [14] was then employed to analyze density of states (DOS), HOMO-LUMO orbitals, hole-electron distributions, charge density difference (CDD), transition density matrix (TDM) map and calculate various parameters involving HOMO-LUMO gap, $E_{S0\text{-}T1}$, $E_{S1\text{-}T1}$, D index, Sr index, etc. Especially, partial density of states (PDOS) was plotted based on the contributions of basis functions to molecular orbitals (MOs) using integrated C-squared population analysis method in Main function 10 of Multiwfn files [14]. Angular momentum S, P, D and defined fragments (e.g., P angular momentum in the tetrapyrrole ring of Ce6 and modeled Gd_2O_3 fragment) were further specified to investigate their effect on the total DOS. All the DOSs at discrete MO energy from -15 to 5 eV were broadened to more intuitively reflect the relations between PDOS using the Gaussian function. The related isosurface maps were rendered with Visual Molecular Dynamics software [15].

Spin-orbit coupling (SOC) matrices were obtained with the ORCA program, using the all-electron calculations through the second-order Douglas-Kroll-Hess (DKH2) method [16]. A segmented all-electron relativistically contracted (SARC) basis set was used for Gd [17] combined with DKH reconstructed versions of def2-TZVP basis sets; and the DKH-def2-TZVP for the rest of the atoms. PBE0 functional with D3BJ correction was selected for calculations. The SARC/J Coulomb fitting basis set was utilized as an auxiliary basis set. For accelerating the calculations, resolution-of-identity-chain-of-sphere exchange (RIJCOSX) [18] approximation was used, and the size of the COSX grid was set as the GridX6 and NoFinalGridX keywords. Solvent effects (water) were considered with the SMD.

1.5 Transient absorption measurement

A regenerative Ti: sapphire amplifier laser with 500 Hz repetition (Legend Elite USP HE+, Coherent, 35 fs, 800 nm) was employed as the primary laser source. The output beam was split into two beams by an optical wedge. One beam with a power of 6 μJ per pulse was focused onto pure water to generate a white light continuum as a probe beam, while the other beam was frequency doubled through a 150 μm BBO crystal to generate a 400 nm pump beam (pulse width: 90–100 fs). The pump beam was passed to an optical delay line to cover the TA experiment from fs to 1 ns (M-ILS200HA, Newport). A mechanical chopper was employed to modulate the pump repetition

frequency to 1/2 the probe repetition rate. The probe beam spot diameter on the sample was ~ 200 μm, while the pump beam was focused to a diameter of ~ 500 μm, with a pump energy of 2 μJ per pulse, covering the probe beam spatially. The probe pulse was recorded using a fiber spectrometer (Avantes, AvaSpec_ULS2048L-USB2) in external trigger mode. The polarization between the pump and probe beam was set to the magic angle (54.7°) with respect to the probe beam.

The sample solutions were placed in a 5-mm-thick quartz cell and stirred magnetically to reduce the photodamage to samples caused by the long-time irradiation in the same position. UV-visible absorption spectra of the samples before and after the experiments showed almost no change. The global fitting analyses based on the singular value decomposition to distinguish the transient species were achieved using the Glotaran software [19].

The triplet TA measurement was conducted by a laser flash photolysis method. The 400 nm frequency-doubled output from a regenerative Ti: sapphire amplifier laser with 500 Hz repetition (Legend Elite USP HE+, Coherent, 35 fs, 800 nm) as excitation source. The analyzing light was derived from a 150 W CW xenon lamp. The excitation pulses and analyzing light beam passed through a 10-mm-thick sample cell with 45° separation angle. The transient signals, passing through a monochromator (WDG30-Z), were amplified by a fast preamplifier with the bandwidth of 350 MHz (SR240A) and recorded on a 300 MHz digital oscilloscope (Tektronix, TDS2024B). Deoxygenated sample solution was prepared using the freeze–pump–thaw technique. Characteristic wavelength kinetics were well fitted with one- or two-exponential function convoluted with a Gaussian response function. The sample concentration is about 3 mg mL^{-1} for CDSNPs complexes, 50 μM for the isolated Ce6 solutions and 30 μM for the isolated MC540 solutions in the fs to μs transient absorption spectra measurements.

1.6 The methods to calculate the lifetimes and quantum yields of triplet state, ISC rate constants, etc.

The triplet lifetimes were acquired using the 440 nm characteristic wavelength kinetics which were fitted with single- or two-exponential function convoluted with instrument response function. The triplet molar absorption coefficient (ε_T) and triplet quantum yield Φ_T can be estimated using the singlet depletion method (Equation 1) and comparative actinometry method (Equation 2) respectively [20]. Furthermore, intersystem crossing rate constant k_{isc} can be obtained using Equation 3.

$$\varepsilon_T = \varepsilon_S \frac{\Delta OD_T}{\Delta OD_S} \tag{1}$$

$$\Phi_T = \Phi_{T(std)} \frac{\Delta OD_T}{\Delta OD_{T(std)}} \frac{\varepsilon_{T(std)}}{\varepsilon_T} \tag{2}$$

$$k_{isc} = \frac{\Phi_T}{\tau_{fl}} \tag{3}$$

wherein, ΔOD_T and ΔOD_S are the triplet absorption maximum and ground-state bleaching minimum, respectively, and ε_S represents the singlet molar absorption coefficient of the corresponding wavelength at ground-state bleaching minimum.

Subscript std denotes standard sample H_2TPP ($\Phi_{T\,(std)}$=0.8, $\varepsilon_{T\,(std)}$≈35000 M^{-1} cm^{-1}).

τ_{fl} is the fluorescence lifetime of the samples. The fluorescence decay curves were recorded using TCSPC method and well fitted with single-exponential function (Figure S8 and S9). In addition, oxygen quenching rate constant k_q^T and proportion of triplet states quenched by oxygen $P_{O_2}^T$ can be obtained according to the Stern–Volmer equation (4) and (5) [21]:

$$k_q^T = \left[\frac{1}{\tau_T} - \frac{1}{\tau_T^0}\right]\frac{1}{[O_2]} \tag{4}$$

$$P_{O_2}^T = \frac{k_q^T[O_2]}{k_T^0 + k_q^T[O_2]} \tag{5}$$

wherein, τ_T and τ_T^0 are the triplet state lifetimes under aerated and deaerated conditions, respectively. $[O_2]$ represents the oxygen concentration, which is about 0.27×10^{-3} M in the aqueous solution at room temperature [22], and k_T^0 (equal to $1/\tau_T^0$) is the decay rate constant of the triplet state under deaerated condition.

1.7 Time-resolved fluorescence measurement

Time-resolved fluorescence spectra at room temperature (298 K) were achieved using a synchroscan streak camera (Hamamatsu C6860, time−resolution 700 fs) coupled to a polychromator. The excitation source of 500 nm laser (with a 35 fs pulse duration and 1 kHz pulse repetition rate) for all samples was produced by a regenerative Ti: sapphire amplifier system (Legend Elite USP HE+, Coherent, Inc., Santa Clara, CA, USA). The overall time resolution was less than 2 ps after fluorescence decay curves were deconvoluted based on a global fitting method. The sample concentration is 1 mg mL^{-1} for CDSNPs@MC540/Ce6 and 0.03 mg L^{-1} for MC540/Ce6. All the decays were re-analysed using the same lifetime parameters to obtain fluorescence decay-associated spectra (FDAS), which are composed of amplitudes of these exponential components as y axis and corresponding emission wavelengths as x axis.

The measured fluorescence intensity F_{exp} can be described as follows:

$$F_{\mathrm{exp}}(t) = f_{\mathrm{pump}} \otimes F_{\mathrm{theo}} = \int f_{\mathrm{pump}}(t) F_{\mathrm{theo}}(t - t')\mathrm{d}t'$$

where f_{pump} represents the pump laser pulse and F_{theo} denotes the theoretical fluorescence intensity which can be considered as a sum of multi-exponential form:

$$F_{\mathrm{theo}}(t) = \sum \varepsilon_i \exp(-t/\tau_i)$$

Herein, the isotropic fluorescence was fitted with a deconvolution procedure based on the global optimization method as expressed as follows:

$$I_n(\lambda,t)=\sum_{i=1}^{n} A_i(\lambda_i)\exp(-t/\tau_i)$$

where τ_i denotes the fluorescence isotropic decay constant and A_i represents the corresponding amplitude.

1.8 ROS generation and live/dead assay of CNE-2 cells for UC-PDT effects of CDSNPs@MC540/Ce6

To confirm the ROS generation in cells, DCFH-DA, as a ROS sensor, was employed to examine the ROS production ability by flow cytometry analysis. Nasopharyngeal carcinoma CNE-2 cells were cultured in the Roswell Park Memorial Institute`s media (RPMI 1640) containing 10% fetal bovine serum, penicillin (100 units per mL), and streptomycin (100 mg mL^{-1}) at 37 °C under 5% CO_2 atmosphere. After growing to logarithmic growth phase, cells were washed by PBS three times and treated with PBS or fresh culture media containing PSs or rare-earth coupled PSs for further 6 h culture. After that, the washed cells were irradiated by a 980 nm laser for 2 min. The experimental and control groups were then cocultured with DCFH-DA solution for another 0.5 h and washed with PBS three times. Then, cells were analyzed by flow cytometry (BD FASAria Cell Sorter, USA). For the photodynamic cytotoxicity assessment, cells treated with samples for 6 h were exposed to a 980 nm laser for 10 min after washed by PBS three times. The excitation light density was set as ~ 0.7 W cm^{-2}. Then cells were washed with PBS twice and stained with 100 μL of PBS containing 2 μM of Calcein-AM and 4.5 μM of propidium iodide (PI) under dark at 37 °C for 15 min. The stained cells were fixed and observed by a laser scanning confocal microscope (Leica TCS SP8 X, Germany) at the green channel (510−560 nm) and red channel (600−680 nm) upon 490 nm excitation.

1.9 Dark cytotoxicity assays

Dark cytotoxicity of CDSNPs@MC540/Ce6 to the normal cell was evaluated using apoptosis analysis of normal liver L-O2 cells stained with the Annexin V-FITC/PI in the presence of samples. L-O2 cells were seeded onto 12-well plates at the density 1×10^6 per well and cultured with Dulbecco's modified Eagle's media (DMEM) containing 10% fetal bovine serum, penicillin (100 units per mL), and streptomycin (100 mg mL^{-1}) at 37 °C under 5% CO_2 for 24 h. Then cells were treated with PBS (negative control), commercial Gd-DTPA, CDSNPs@MC540, CDSNPs@Ce6 and CDSNPs@(MC540 + Ce6) in dark for another 48 h. Cells were collected by the trypsin digestion method after washed with PBS twice. Cold 70% ethanol was added and gently shaken to disperse cells well after removing the supernatant. The solution then incubated for 30 min at 20 °C. Ethanol was washed off by centrifugation twice with PBS. Cells were then resuspended in 195 μL of PBS, adding 5 μL of Annexin V-FITC and photophobically incubated for 10 min at room temperature. Cells were resuspended along with 10 μL PI (20 μg mL^{-1}) after washed with PBS twice. At last, a FACScan flow cytometer (BD C6, Becton Dickinson) was employed to analyze apoptosis of L-O2 cells.

2. Supplementary figures

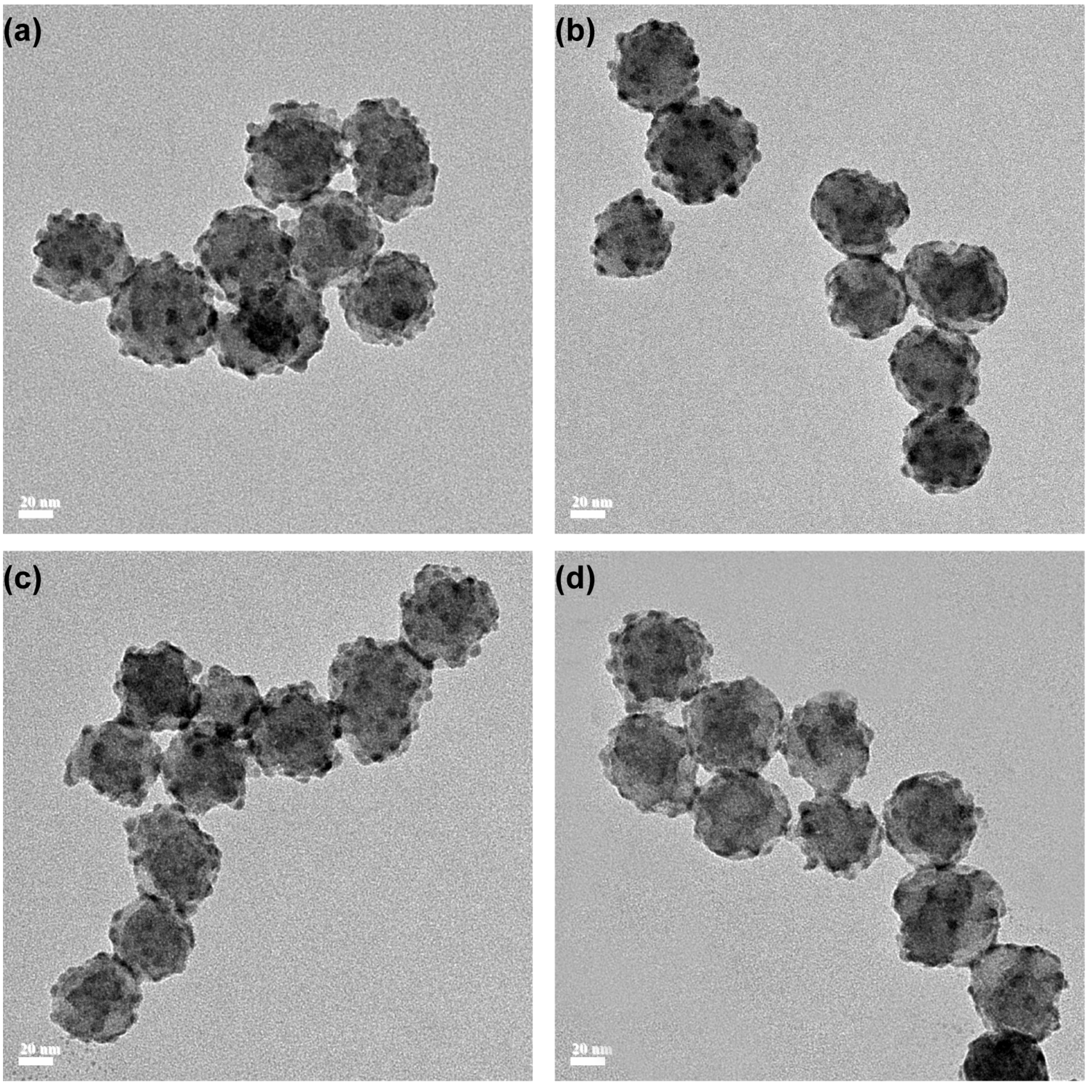

Figure S1. Transmission electronic microscope (TEM) images of CDSNPs dispersed in DMSO before (a) and after (c) 120 h as well as in aqueous saline solutions before (b) and after (d) 120 h.

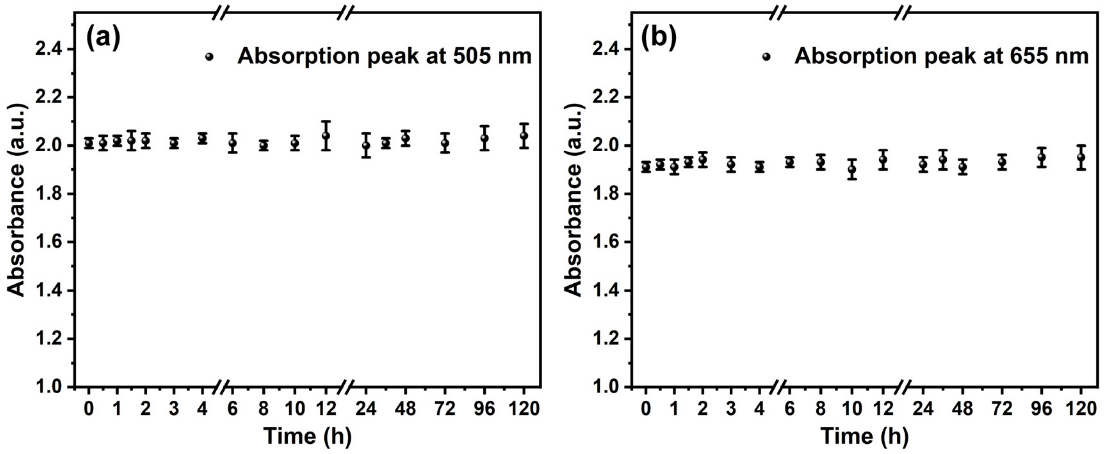

Figure S2. Photonic stability validated by the absorbance changes versus time monitored at 505 nm (a) and 655 nm (b).

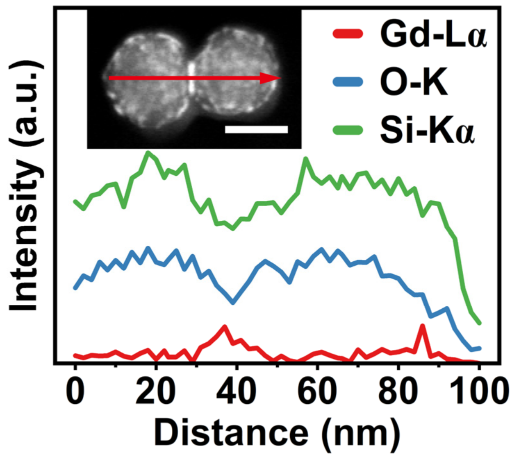

Figure S3. EDX elemental line scanning curves. Insert micrograph denotes the line scanning position (scale bar: 50 nm).

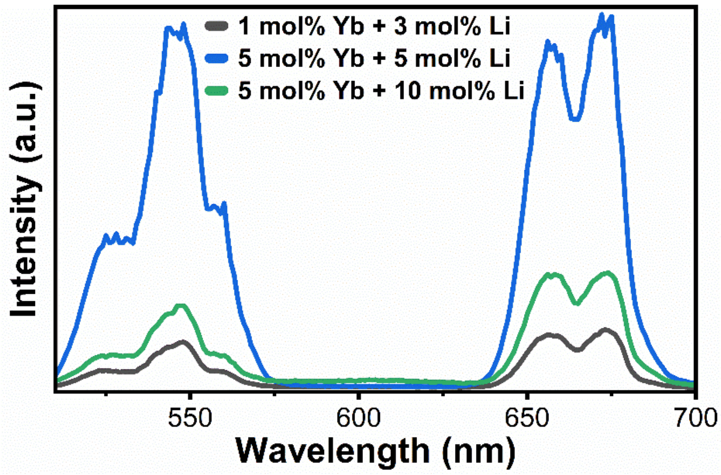

Figure S4. Upconversion luminescence spectra of SiO_2@Gd_2O_3:Yb^{3+}/2%Er^{3+}/Li^{+} CDSNPs with different Yb^{3+} and Li^{+} doping concentrations in PBS under the 980nm laser excitation.

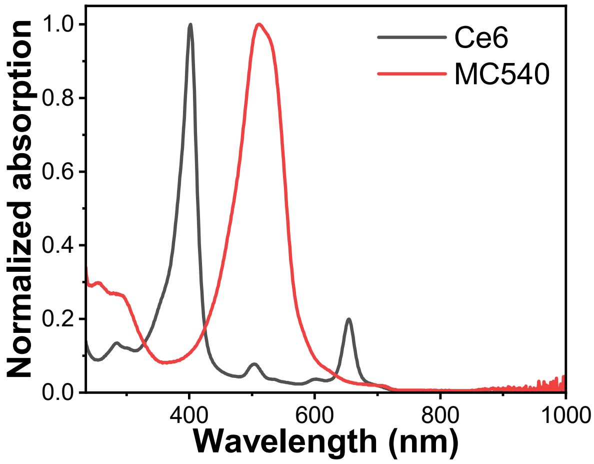

Figure S5. UV-Vis-NIR absorption spectra of Ce6 and MC540 in PBS.

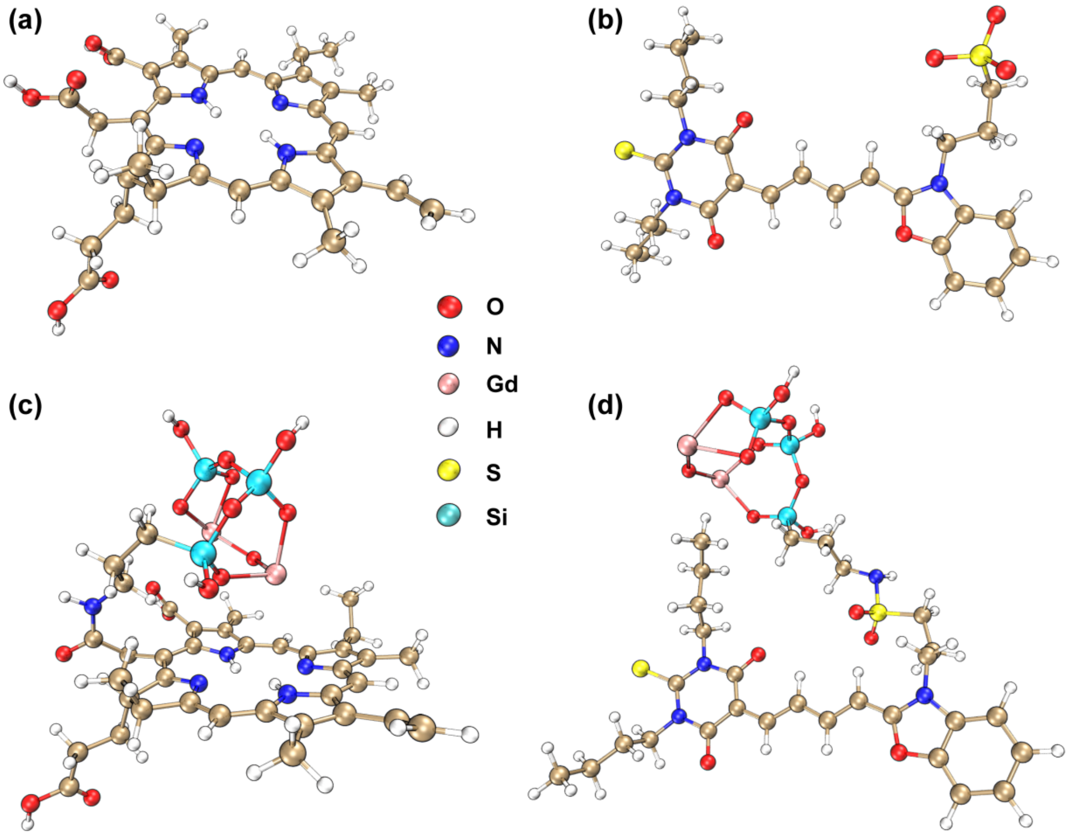

Figure S6. Global energy minimum structures obtained in the conformational searching for Ce6 (a) and MC540 (b). Constructed models for CDSNPs@Ce6 (c) and CDSNPs@MC540 (d) based on the most stable structures of MC540 and Ce6.

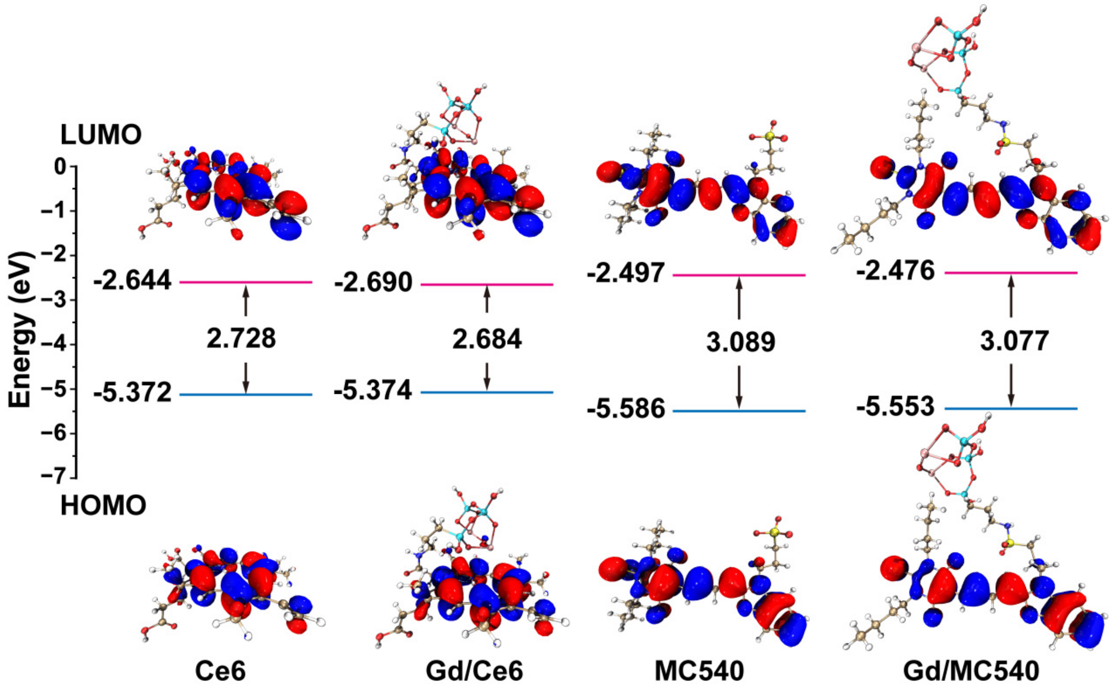

Figure S7. The orbital energy and distribution of HOMOs and LUMOs for Ce6, Gd/Ce6, MC540 and Gd/MC540.

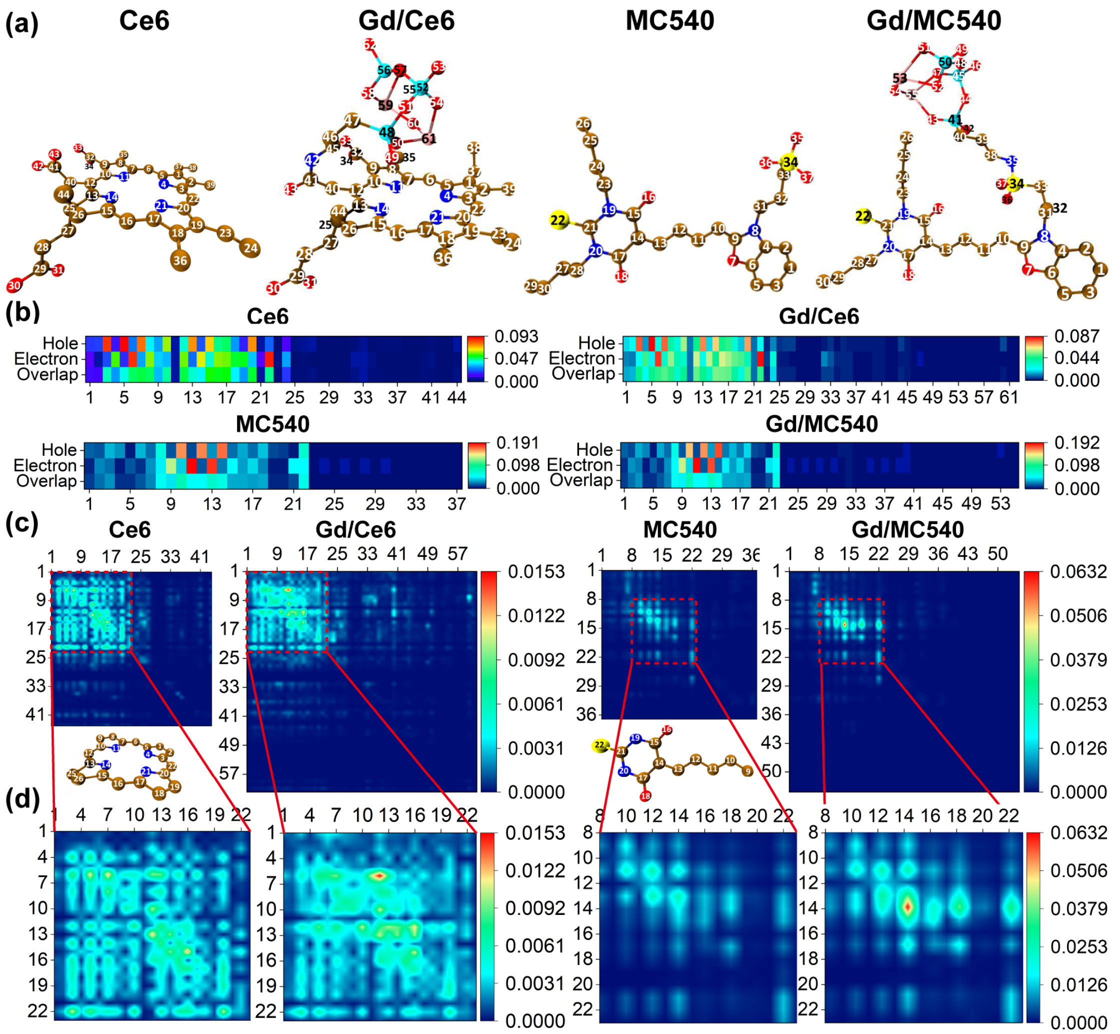

Figure S8. (a) Geometry structures labelled with non-hydrogen atoms order (b) The electron-hole heat maps exhibit the contribution of labelled non-hydrogen atoms to the distributions of hole, electron and their overlap. (c) Graphical representation of transition density matrix (TDM) at the S_1 state. The red rectangles denote the main hole-electron localization as fragments shown below the TDM graphs for Ce6 and Gd/Ce6 as well as MC540 and Gd/MC540. (d) Enlarged TDM regions showing the clearer details.

For a more detailed illustration of S_1 electronic excitation process, hole and electron heat maps and the transition density matrix (TDM) contributed by specific atoms are calculated to analyze the hole-electron localization and the transition probability for the $S_0 \rightarrow S_1$ transition. Due to minor contribution to electronic excitation, the influence of hydrogen atoms was neglected. The optimized geometry structures are labeled with non-hydrogen atom numberings to classify the contribution of atoms to the hole-electron heat map and TDM map (Figure S8(a)). Detailed contributions by specific atoms are documented in Table S1 and S2. In view of both Ce6 and Gd/Ce6, atoms numbered 1-22 contribute more than 90% to both hole and electron distributions, confirming the tetrapyrrole ring as main excitation region (Figure S8(b)). The TDM graphs also indicates predominant electron-hole distributions on the whole tetrapyrrole ring fragment (Figure S8(c)). The high

symmetry along the diagonal is attributed to the delocalized property of the conjugated system, as presented in Figure S8(d). In comparison, TDM pattern for Gd/Ce6 exhibits larger non-diagonal elements below diagonal, indicating electrons in tetrapyrrole ring fragment are more inclined to transition to atoms with lower numbering, which confirms an ever-growing charge transfer induced by the rare earth fragment, consistent with the findings from the hole-electron analysis. Regarding MC540 and Gd/MC540, atoms labeled with numbers 9-22 contribute over 89.5% to both hole and electron distributions. TDM graph of Gd/MC540 revealed larger TDM elements from atoms 9 to 22 to atoms C14 and C17 compared with MC540, as indicated in Figure S8(d). It seems electrons tend to transfer towards moiety close to rare earth Gd, in both Ce6 and MC540 when coupled with the rare earth Gd fragment.

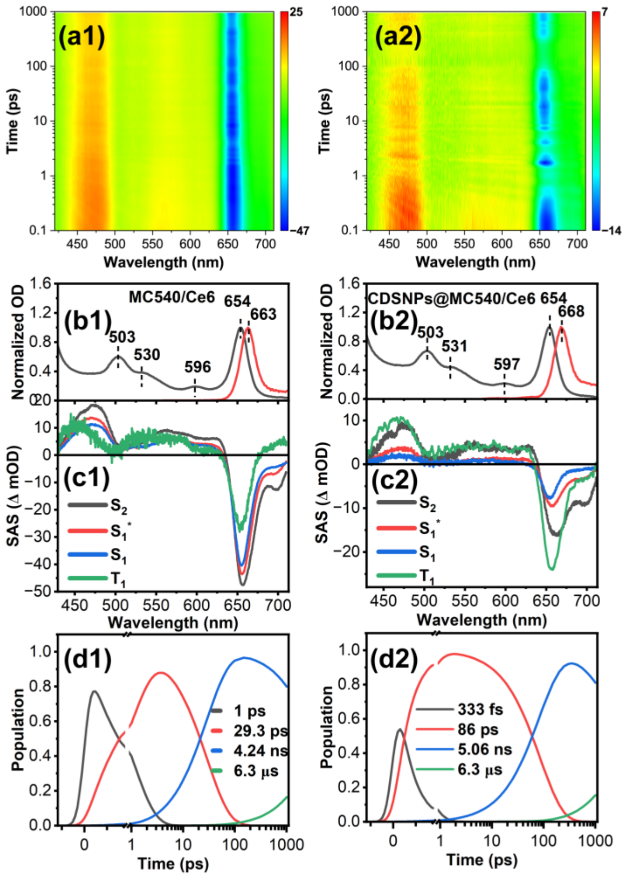

Figure S9. Transient absorption contour maps (a1-a2), ground-state absorption (black) and fluorescence spectra (red) (b1-b2), species associated spectra (c1-c2) and population evolution (d1-d2) of MC540/Ce6 and CDSNPs@MC540/Ce6 in PBS in the range from femtosecond to nanosecond time scale. Pump wavelength is 400 nm.

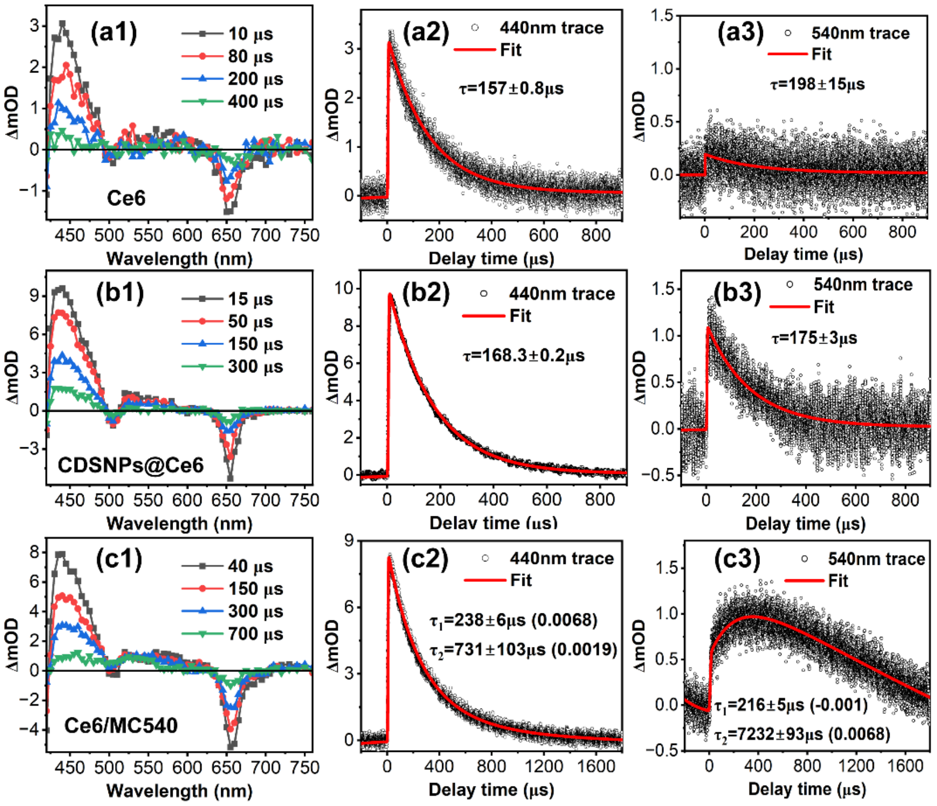

Figure S10. Triplet transient absorption spectra (a1-c1) and characteristic wavelength kinetics at 440 nm (a2-c2) and 540 nm (a3-c3) of Ce6, CDSNPs@Ce6 and Ce6/MC540 in deaerated PBS. Pump wavelength is 400 nm. Red solid lines are the corresponding single- or two-exponential fits; Fitting results are shown in the form of Lifetime values ± standard deviation. Numbers in parentheses denote the amplitudes.

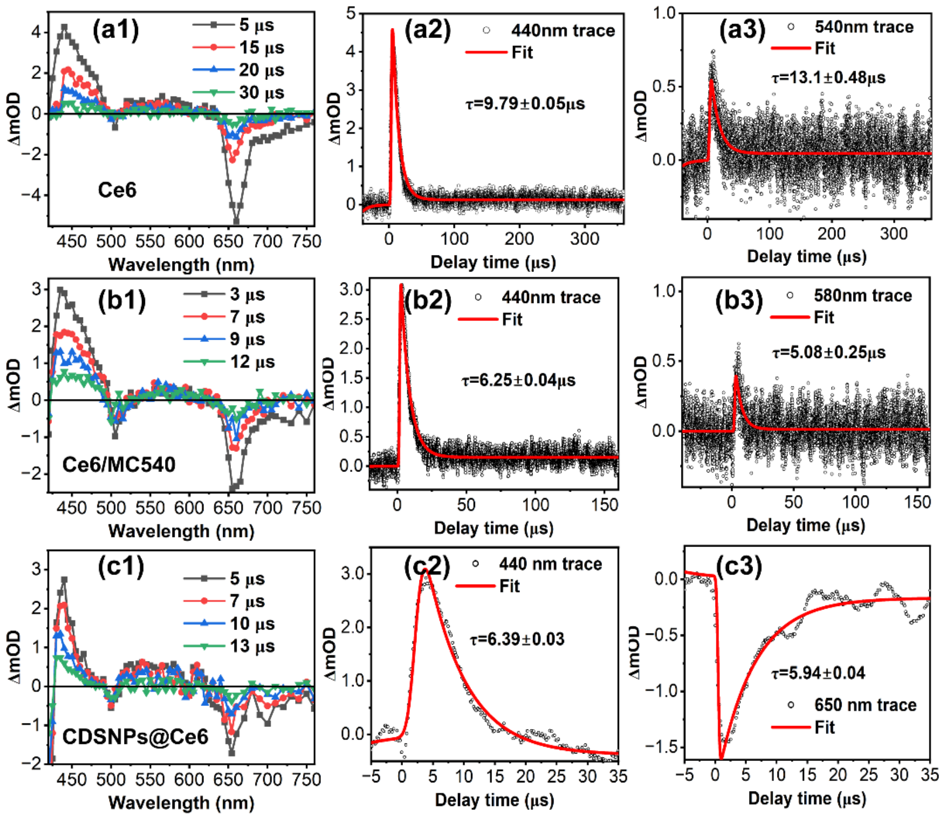

Figure S11. Triplet transient absorption spectra (a1-c1) and characteristic wavelength kinetics at 440 nm (a2-c2) and 540 nm (a3-c3) of Ce6, Ce6/MC540 and CDSNPs@Ce6 in aerated PBS. Pump wavelength is 400 nm. Red solid lines are the corresponding single- or two-exponential fits; Fitting results are shown in the form of Lifetime values ± standard deviation. Numbers in parentheses denote the amplitudes.

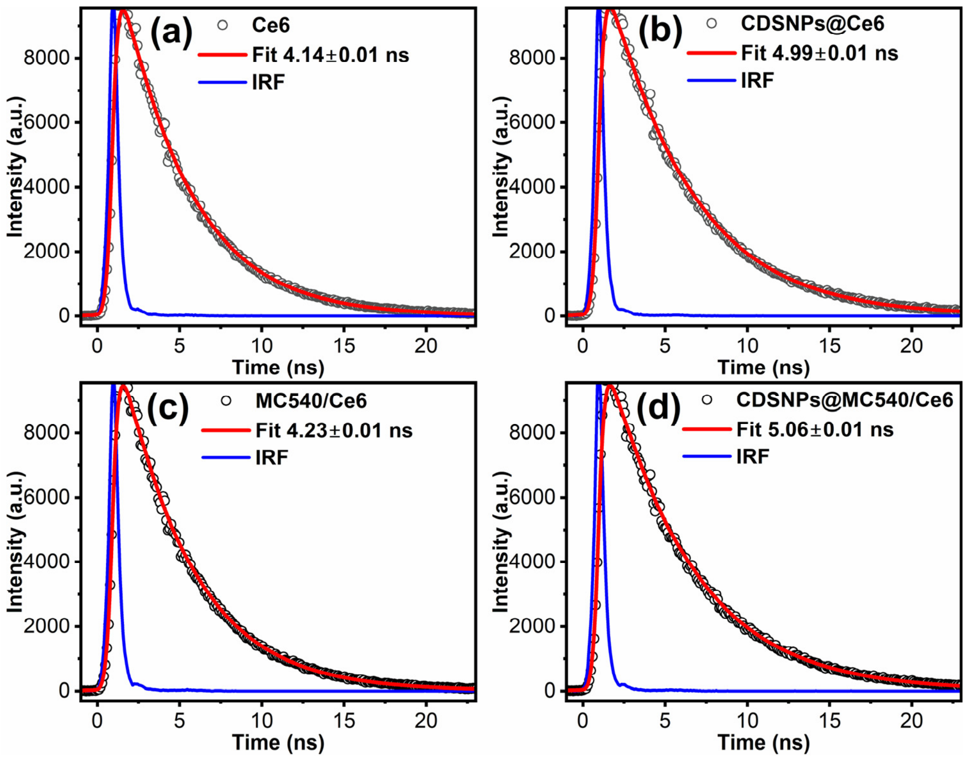

Figure S12. Fluorescence decay traces for Ce6 (a), CDSNPs@Ce6 (b), MC540/Ce6 (c) and CDSNPs@MC540/Ce6 in PBS, recorded at 662 nm. The excitation wavelength was 400 nm. Black open circles are observed data points; Red solid lines are the corresponding single-exponential fits; Blue solid lines represent instrument response function (IRF); Fitting results are shown in the form of Lifetime values ± standard deviation.

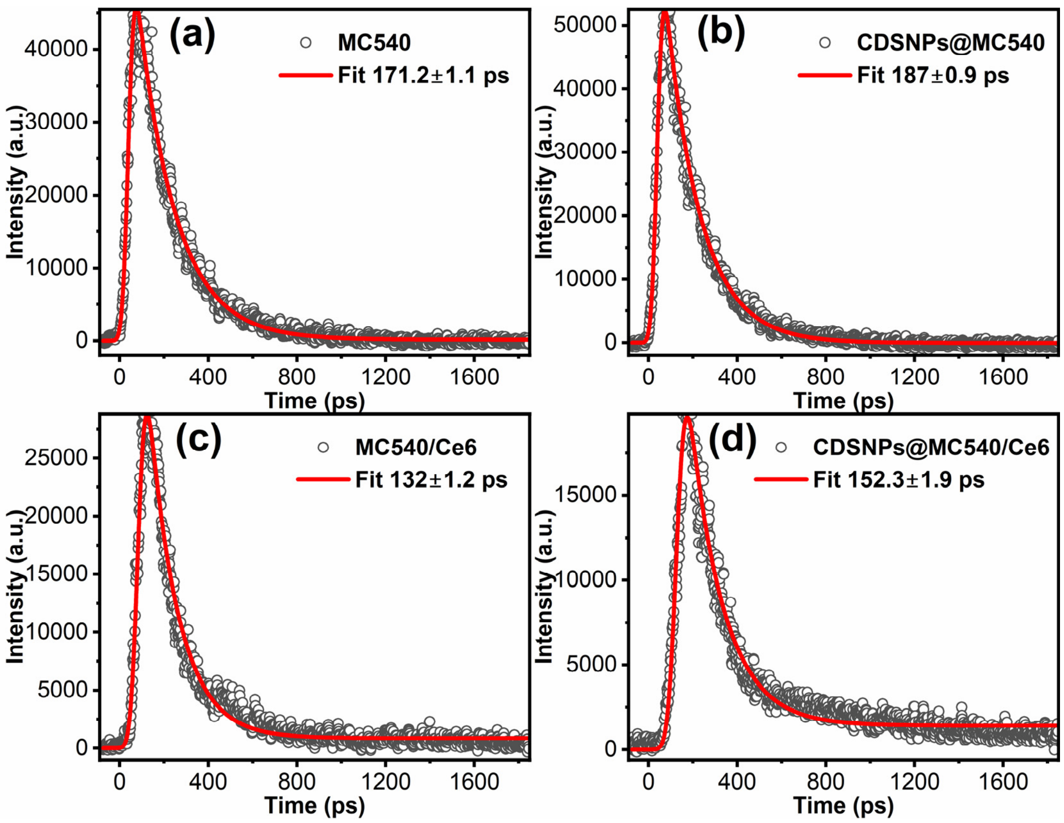

Figure S13. Fluorescence decay traces for MC540 (a), CDSNPs@MC540 (b), MC540/Ce6 (c) and CDSNPs@MC540/Ce6 in PBS, recorded at 576 nm. The excitation wavelength was 500 nm. Black open circles are observed data points; Red solid lines are the corresponding single-exponential fits; Fitting results are shown in the form of Lifetime values ± standard deviation.

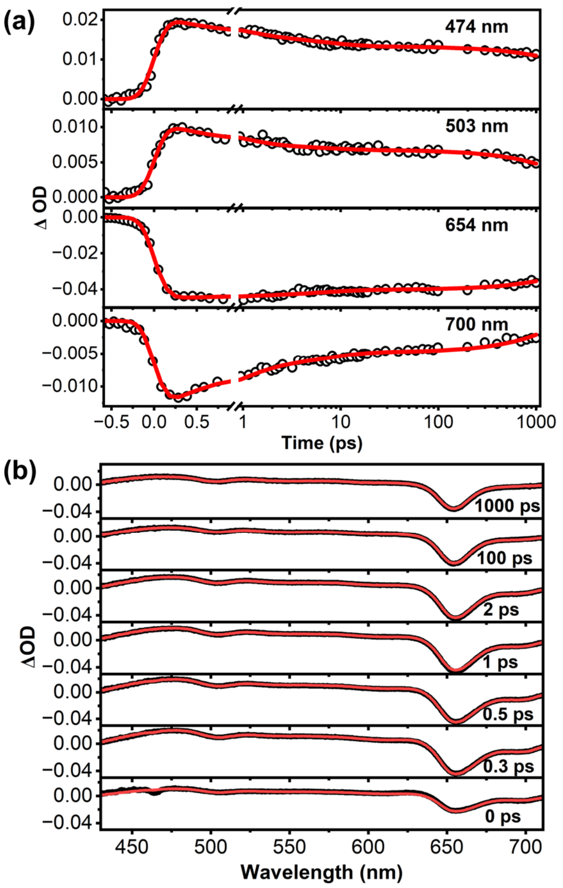

Figure S14. (a) Time profiles at several selected wavelengths of Ce6 in PBS after 400 nm (90-100 fs) pump. Red solid lines are the fitting results obtained from global target analysis. (b) Transient absorption spectra at different times of Ce6; solid red lines are results of global target analysis.

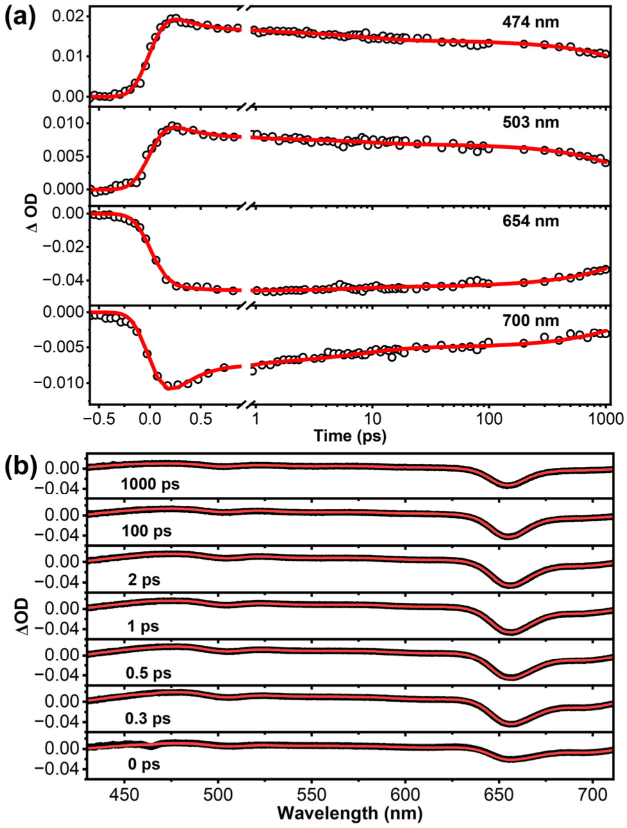

Figure S15. (a) Time profiles at several selected wavelengths of CDSNPs@Ce6 in PBS after 400 nm (90-100 fs) pump. Red solid lines are the fitting results obtained from global target analysis. (b) Transient absorption spectra at different times of CDSNPs@Ce6; solid red lines are results of global target analysis.

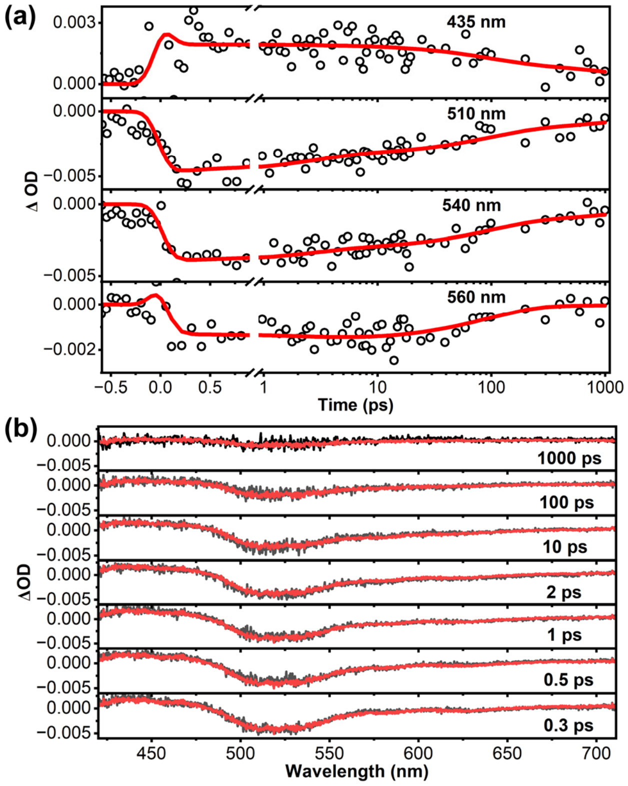

Figure S16. (a) Time profiles at several selected wavelengths of MC540 in PBS after 400 nm (90-100 fs) pump. Red solid lines are the fitting results obtained from global analysis based on sequential model. (b) Transient absorption spectra at different times of MC540; solid red lines are results of global analysis.

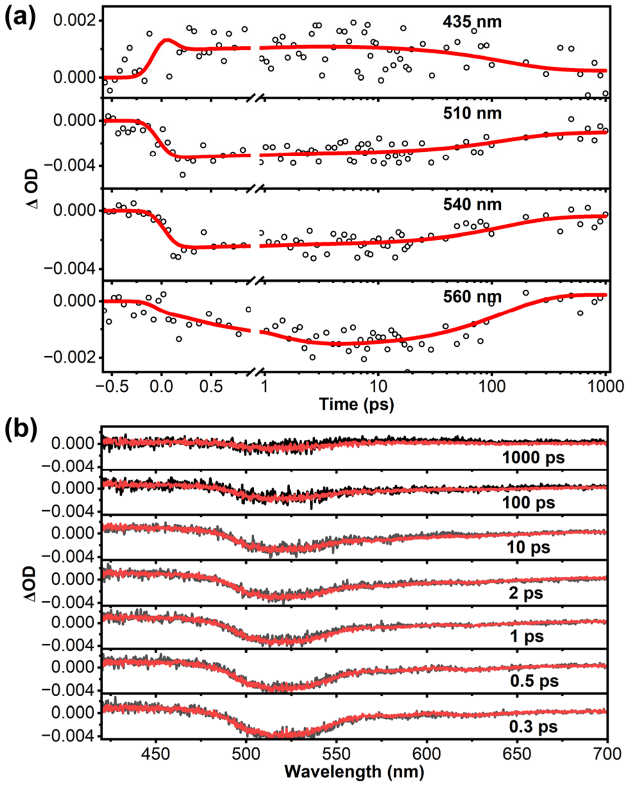

Figure S17. (a) Time profiles at several selected wavelengths of CDSNPs@MC540 in PBS after 400 nm (90-100 fs) pump. Red solid lines are the fitting results obtained from global analysis based on sequential model. (b) Transient absorption spectra at different times of CDSNPs@MC540; solid red lines are results of global analysis.

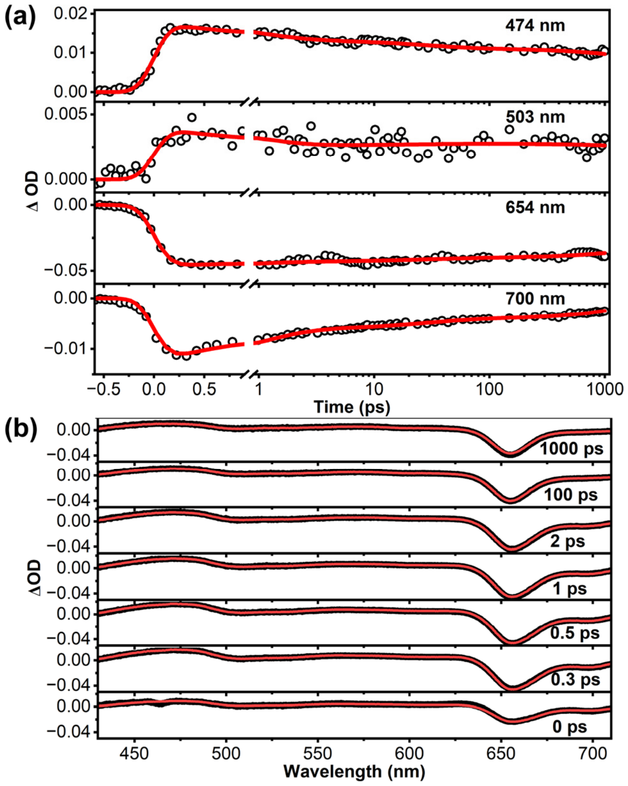

Figure S18. (a) Time profiles at several selected wavelengths of MC540/Ce6 in PBS after 400 nm (90-100 fs) pump. Red solid lines are the fitting results obtained from global target analysis. (b) Transient absorption spectra at different times of MC540/Ce6; solid red lines are results of global target analysis.

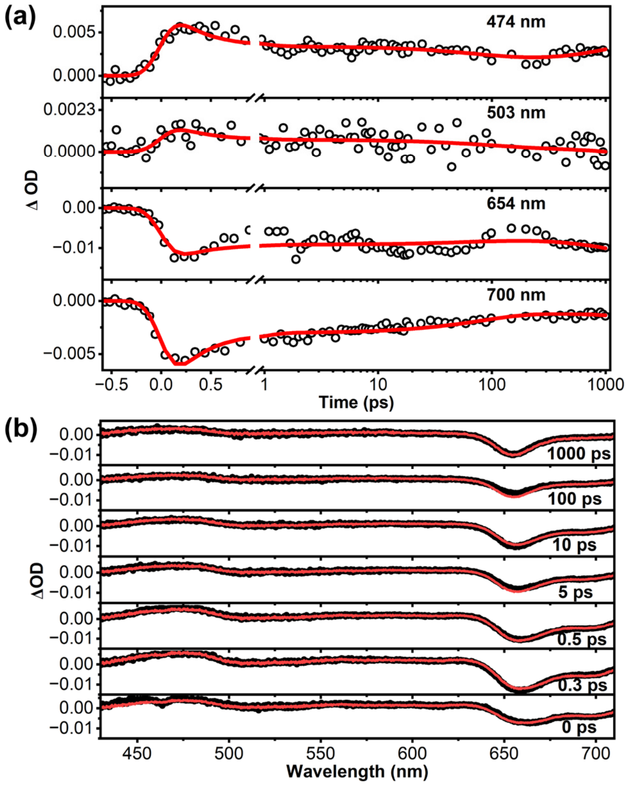

Figure S19. (a) Time profiles at several selected wavelengths of CDSNPs@MC540/Ce6 in PBS after 400 nm (90-100 fs) pump. Red solid lines are the fitting results obtained from global target analysis. (b) Transient absorption spectra at different times of CDSNPs@MC540/Ce6; solid red lines are results of global target analysis.

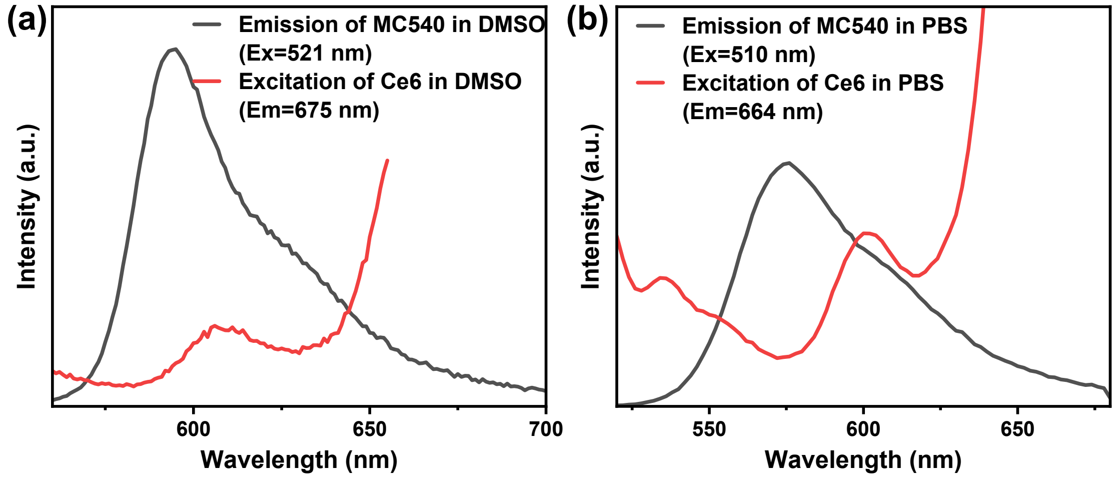

Figure S20. (a) Spectra overlap between emission spectrum of MC540 at the excitation wavelength of 521 nm and excitation spectrum of Ce6 monitoring 675 nm emission in DMSO solvent. (b) Spectra overlap between emission spectrum of MC540 at the excitation wavelength of 510 nm and excitation spectrum of Ce6 detecting 664 nm emission in PBS solvent.

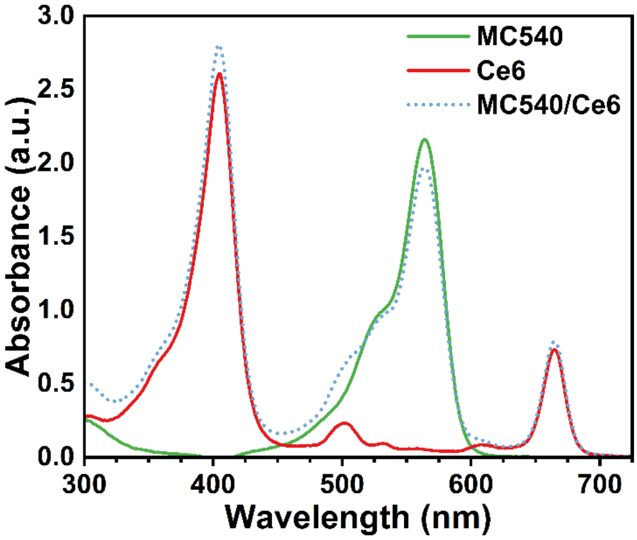

Figure S21. Absorption spectra of MC540, Ce6 and MC540/Ce6 in DMSO.

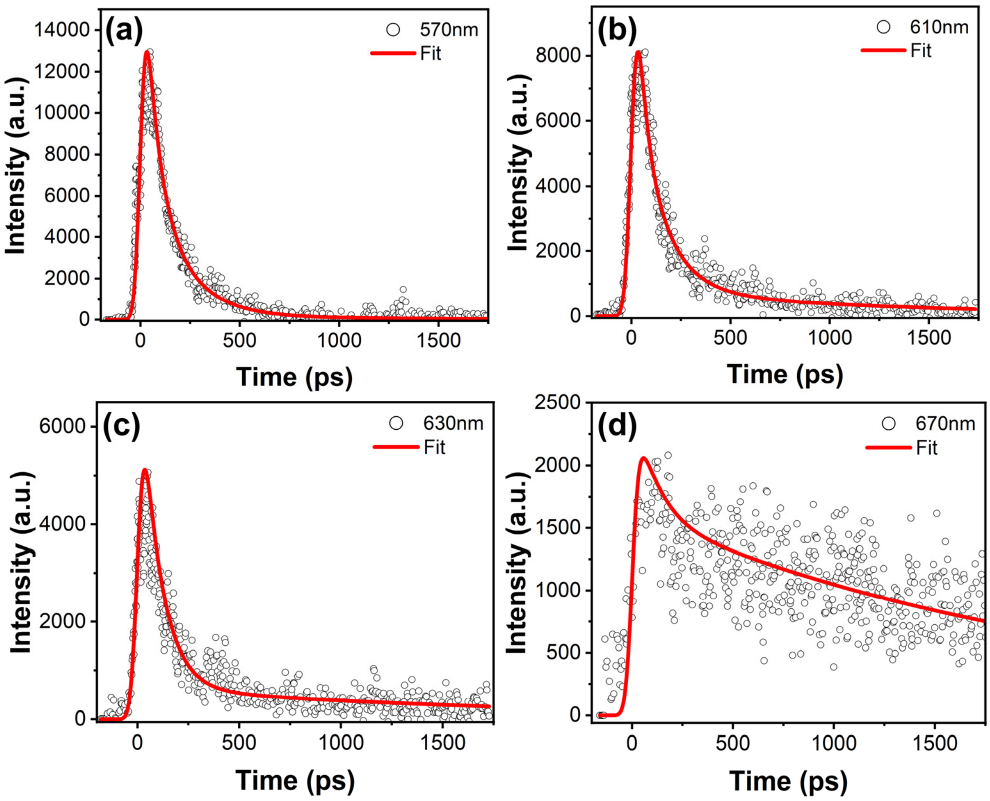

Figure S22. Fluorescence decay curves (hollow circles) and fitting results (red lines) of MC540/Ce6 at selected wavelength (λ_{ex}=500nm).

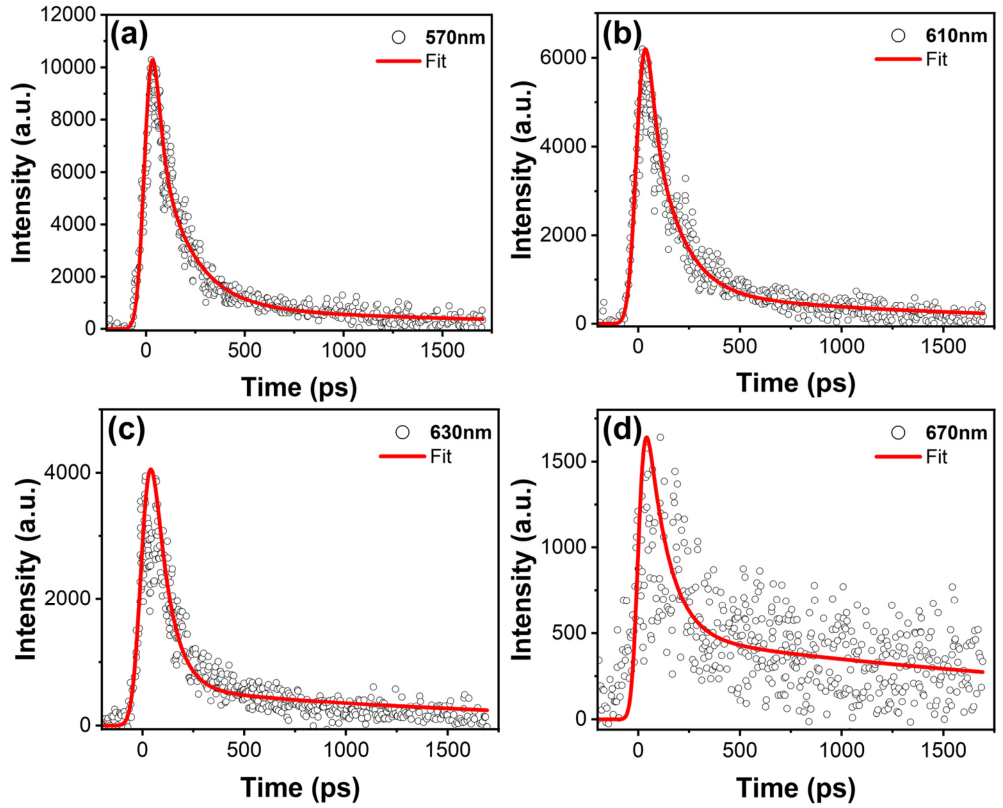

Figure S23. Fluorescence decay curves (hollow circles) and fitting results (red lines) of Gd@MC540/Ce6 at selected wavelength (λ_{ex}=500nm).

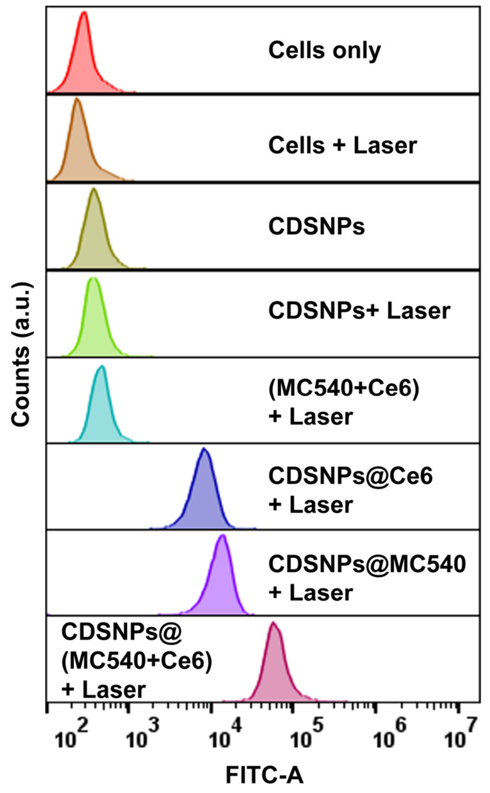

Figure S24. ROS generation capacity investigated using flow cytometry techniques.

ROS generation *in vitro* can be confirmed using flow cytometry analysis. In this

experiment, DCFH-DA, as a most widely used ROS probe, can be rapidly oxidized by intracellular ROS species to transform into the form of DCF molecule emitting green fluorescence. According to the detected fluorescence intensity by the flow cytometry, one can quantitatively assess the amount of reactive oxygen species. The experimental results demonstrate that both CDSNPs@Ce6 and CDSNPs@MC540 possess the good ROS generation capacity in comparison to the control group (Figure S24). Moreover, dual-PS loaded CDSNPs exhibit much higher than 2-fold of fluorescence compared to any single-PS CDSNPs, further confirming the synergistic photodynamic effect of the dual-PS UC-PDT system.

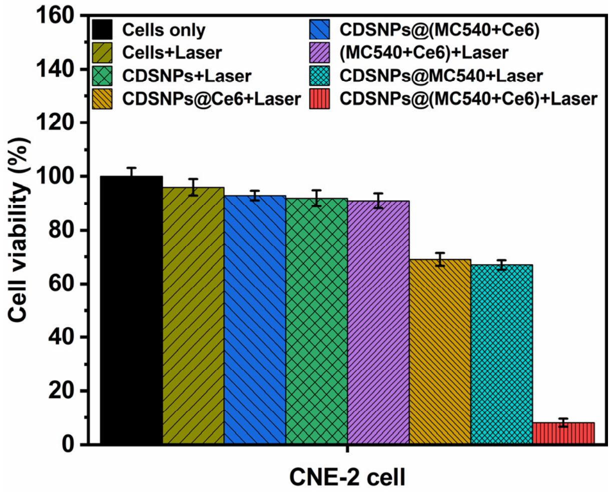

Figure S25. The quantitative comparison of the cell viability in live/dead cell assay.

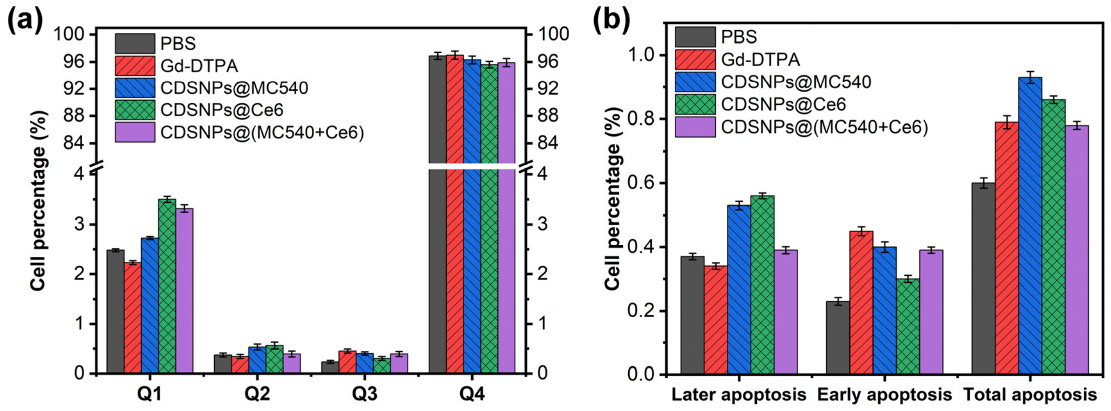

Figure S26. (a), (b) Statistical results of apoptosis assay of L-O2 cells incubated with PBS, commercial Gd-DTPA, CDSNPs@MC540, CDSNPs@Ce6 and CDSNPs@(MC540+Ce6) with 20 μg mL^{-1} for 48 h. Q1 is the region where cells should not turn up; Q2 and Q3 denote the apoptosis cells in the later and early stages, respectively; and Q4 represents the normal living cells.

3. Supplementary tables

Table S1. Contributions of all the non-hydrogen atoms of Ce6 and Gd/Ce6 to hole, electron and their overlap in the real space.

Atom	Overlap/%	Electron/%	Hole/%	Overlap/%	Electron/%	Hole/%
		Ce6			Gd/Ce6	
1C	1.426	1.095	1.857	1.517	1.186	1.941
2C	1.326	0.892	1.971	1.552	0.997	2.417
3C	4.283	2.164	8.48	4.345	2.416	7.814
4N	2.976	5.588	1.585	2.596	4.844	1.391
5C	4.125	1.843	9.23	4.26	2.093	8.671
6C	4.767	8.648	2.627	4.768	8.21	2.769
7C	4.905	3.071	7.835	5.029	3.246	7.791
8C	4.818	5.95	3.901	4.654	5.567	3.891
9C	3.252	3.489	3.031	3.232	3.611	2.893
10C	4.488	3.333	6.043	4.287	3.219	5.71
11N	0.838	0.912	0.771	0.964	0.94	0.989
12C	4.94	5.852	4.17	5.149	5.993	4.424
13C	4.278	2.459	7.444	4.315	2.515	7.405
14N	3.817	6.463	2.255	3.404	5.946	1.949
15C	5.367	4.171	6.906	5.385	4.285	6.767
16C	5.19	5.927	4.545	5.248	6.174	4.461
17C	5.285	5.808	4.81	5.182	5.696	4.716
18C	3.883	5.052	2.984	3.914	5.113	2.996
19C	2.921	5.137	1.661	2.901	4.926	1.708
20C	5.06	3.432	7.46	5.03	3.528	7.173
21N	0.923	0.985	0.864	0.917	0.96	0.875
22C	4.592	8.803	2.396	4.682	8.508	2.576
23C	0.2	0.256	0.156	0.323	0.471	0.221
24C	1.923	3.047	1.213	1.859	2.999	1.153
25C	0.192	0.11	0.337	0.267	0.166	0.429
26C	0.216	0.131	0.356	0.315	0.197	0.505
27C	0.248	0.106	0.578	0.223	0.092	0.541
28C	0.049	0.031	0.078	0.009	0.001	0.079
29C	0.009	0.006	0.012	0.013	0.012	0.014
30O	0.002	0.001	0.004	0.002	0.001	0.004
31O	0.003	0.001	0.008	0.002	0.001	0.005
32C	0.324	0.849	0.124	0.497	1.175	0.21
33O	0.541	0.695	0.421	0.487	0.652	0.364
34O	0.158	0.261	0.096	0.147	0.241	0.089
35C	0.118	0.091	0.154	0.236	0.232	0.24
36C	0.107	0.095	0.12	0.219	0.264	0.181
37C	0.068	0.026	0.176	0.102	0.075	0.141
38C	0.005	0.004	0.008	0.071	0.046	0.111

39C	0.046	0.02	0.103	0.063	0.024	0.167
40C	0.147	0.22	0.099	0.176	0.154	0.202
41C	0.199	0.247	0.161	0.161	0.206	0.126
42O/N	0.026	0.031	0.022	0.03	0.045	0.02
43O	0.042	0.046	0.038	0.038	0.04	0.035
44C	0.288	0.158	0.524	0.237	0.125	0.449
Total	100	100	100			
45C				0.14	0.143	0.138
46C				0.001	0	0.006
47C				0	-0.002	0
48Gd				0.009	0.009	0.009
49O				0.006	0.004	0.011
50O				0.01	0.004	0.022
51O				0.001	0.001	0.001
52Gd				0.001	0.001	0.002
53O				0	0	0
54O				0.003	0.002	0.006
55Gd				0	0	0
56Gd				0	0	0
57O				0.003	0.001	0.019
58O				0.001	0.001	0.001
59Si				0.054	0.045	0.065
60O				0.115	0.023	0.577
61Si				0.064	0.057	0.071
62O				0	0	0
Total				100	100	100

Table S2. Contributions of all the non-hydrogen atoms of MC540 and Gd/MC540 to hole, electron and their overlap in the real space.

Atom	Overlap/%	Electron/%	Hole/%	Overlap/%	Electron/%	Hole/%
		MC540			Gd/MC540	
1C	1.148	0.702	1.876	1.127	0.735	1.729
2C	1.937	2.58	1.455	1.93	2.696	1.382
3C	2.692	2.082	3.48	2.673	2.188	3.267
4C	0.948	0.332	2.703	0.898	0.328	2.454
5C	0.845	1.604	0.446	0.832	1.686	0.411
6C	1.616	0.738	3.539	1.528	0.736	3.173
7O	2.4	2.694	2.139	2.417	2.847	2.052
8N	5.437	4.731	6.248	5.346	4.945	5.779
9C	4.494	12.957	1.559	4.727	13.435	1.663
10C	8.047	4.033	16.056	8.051	4.238	15.296
11C	5.037	19.104	1.328	4.995	19.184	1.301
12C	5.054	1.565	16.321	5.146	1.625	16.301
13C	7.086	18.016	2.787	7.209	17.549	2.961

14C	6.103	2.206	16.887	5.882	1.966	17.597
15C	3.202	4.833	2.121	3.257	4.688	2.263
16O	3.181	3.097	3.266	3.245	2.998	3.513
17C	2.923	4.213	2.028	3.013	4.087	2.221
18O	3.135	2.574	3.819	3.195	2.457	4.156
19N	0.638	0.319	1.274	0.631	0.301	1.321
20N	0.552	0.306	0.996	0.555	0.295	1.044
21C	1.407	4.819	0.411	1.448	4.53	0.463
22S	5.823	4.919	6.893	5.939	4.803	7.342
23C	0.027	0.006	0.123	0.005	0	0.128
24C	0	-0.052	0.106	0	-0.033	0.088
25C	0.013	0.008	0.022	0.019	0.012	0.029
26C	0	-0.001	0.011	0	-0.003	0.007
27C	0.031	0.009	0.108	0.023	0.004	0.128
28C	0	-0.07	0.095	0	-0.07	0.093
29C	0.017	0.015	0.019	0.017	0.016	0.018
30C	0	-0.004	0.011	0	-0.005	0.01
31C	0.041	0.008	0.215	0.199	0.157	0.254
32C	0.019	0.012	0.031	0.389	0.362	0.418
33C	0.003	0.004	0.003	0.074	0.067	0.082
34S	0.003	0.004	0.002	0.01	0.014	0.008
35O/N	0.003	0.001	0.008	0	-0.001	0
36O	0.012	0.004	0.04	0.003	0.003	0.004
37O	0.002	0.001	0.003	0	-0.002	0.006
Total	100	100	100			
38C				0.004	0.008	0.002
39C				0	-0.006	0
40C				0	-0.002	-0.001
41Gd				0	0.001	0
42O				0	0	0
43O				0	0	0
44O				0	0	0
45Gd				0	0	0
46O				0	0	0
47O				0	0	0
48O				0	0	0
49O				0	0	0
50Gd				0	0	0
51O				0	0	0
52O				0	0	0
53Si				0	0	-0.001
54O				0	0	0.001
55Si				0	0	0.001
Total				100	100	100

4. References

[1] J. Yin, X. Wang, H. Zheng *et al.*, "Silica nanoparticles decorated with gadolinium oxide nanoparticles for magnetic resonance and optical imaging of tumors," *ACS Appl. Nano Mater.,* vol. 4, no. 4, pp. 3767-3779, 2021.

[2] T. Entradas, S. Waldron, and M. Volk, "The detection sensitivity of commonly used singlet oxygen probes in aqueous environments," *J. Photochem. Photobiol., B,* vol. 204, p. 111787, 2020.

[3] C. Bannwarth, E. Caldeweyher, S. Ehlert *et al.*, "Extended tight - binding quantum chemistry methods," *Wires. Comput. Mol. Sci.,* vol. 11, no. 2, p. e1493, 2021.

[4] C. Bannwarth, S. Ehlert, S. Grimme, and B. P. S.-C. Tight-Binding, "GFN2-xTB-An Accurate and Broadly Parametrized Self-Consistent Tight-Binding Quantum Chemical Method with Multipole Electrostatics and Density-Dependent Dispersion Contributions," *J. Chem. Theory Comput.,* vol. 15, no. 3, pp. 1652-1671, 2019.

[5] S. Grimme, S. Ehrlich, and L. Goerigk, "Effect of the damping function in dispersion corrected density functional theory," *J. Comput. Chem.,* vol. 32, no. 7, pp. 1456-1465, 2011.

[6] C. M. Porto, L. C. Santana, and N. H. Morgon, "Theoretical investigation of the cooperative effect of solvent: a case study," *Phys. Chem. Chem. Phys.,* vol. 24, no. 23, pp. 14603-14615, 2022.

[7] L. Goerigk and S. Grimme, "Efficient and Accurate Double-Hybrid-Meta-GGA Density Functionals Evaluation with the Extended GMTKN30 Database for General Main Group Thermochemistry, Kinetics, and Noncovalent Interactions," *J. Chem. Theory Comput.,* vol. 7, no. 2, pp. 291-309, 2011.

[8] F. Weigend and R. Ahlrichs, "Balanced basis sets of split valence, triple zeta valence and quadruple zeta valence quality for H to Rn: Design and assessment of accuracy," *Phys. Chem. Chem. Phys.,* vol. 7, no. 18, pp. 3297-3305, 2005.

[9] F. Neese, F. Wennmohs, U. Becker, and C. Riplinger, "The ORCA quantum chemistry program package," *J. Chem. Phys.,* vol. 152, no. 22, p. 224108, 2020.

[10] "T. Lu, Molclus program, Version 1.9.9.9, http://www.keinsci.com/research/molclus.html (accessed 12, August, 2022)." (accessed.

[11] T. Yanai, D. P. Tew, and N. C. Handy, "A new hybrid exchange–correlation functional using the Coulomb-attenuating method (CAM-B3LYP)," *Chem. Phys. Lett.,* vol. 393, no. 1-3, pp. 51-57, 2004.

[12] C. Adamo and V. Barone, "Toward reliable density functional methods without adjustable parameters: The PBE0 model," *J. Chem. Phys.,* vol. 110, no. 13, pp. 6158-6170, 1999.

[13] D. Andrae, U. Häußermann, M. Dolg, H. Stoll, and H. Preuß, "Energy-adjustedab initio pseudopotentials for the second and third row transition elements," *Theor. Chim. Acta,* vol. 77, no. 2, pp. 123-141, 1990.

[14] T. Lu and F. Chen, "Multiwfn: A multifunctional wavefunction analyzer," *J. Comput. Chem.,* vol. 33, no. 5, pp. 580-592, 2012.

[15] W. Humphrey, A. Dalke, and K. Schulten, "VMD: visual molecular dynamics," *J. Mol. Graph.,* vol. 14, no. 1, pp. 33-38, 1996.

[16] T. Nakajima and K. Hirao, "The Douglas–Kroll–Hess Approach," *Chem. Rev.,* vol. 112, no. 1, pp. 385-402, 2012.

[17] D. Aravena, F. Neese, and D. A. Pantazis, "Improved Segmented All-Electron Relativistically Contracted Basis Sets for the Lanthanides," *J. Chem. Theory Comput.,* vol. 12, no. 3, pp.

1148-1156, 2016.

[18] B. Helmich-Paris, B. de Souza, F. Neese, and R. Izsák, "An improved chain of spheres for exchange algorithm," *J. Chem. Phys.,* vol. 155, no. 10, p. 104109, 2021.

[19] J. J. Snellenburg, S. Laptenok, R. Seger, K. M. Mullen, and I. H. M. van Stokkum, "Glotaran: A Java-Based Graphical User Interface for the R Package TIMP," *J. Stat. Softw.,* vol. 49, no. 3, pp. 1 - 22, 2012.

[20] I. Carmichael and G. L. Hug, "Triplet-triplet absorption spectra of organic molecules in condensed phases," *J. Phys. Chem. Ref. Data,* vol. 15, pp. 1-250, 1986.

[21] A. M. M. Alazaly, G. J. Clarkson, M. D. Ward, and A. A. Abdel-Shafi, "Mechanism of Oxygen Quenching of the Excited States of Heteroleptic Chromium(III) Phenanthroline Derivatives," *Inorg. Chem.,* vol. 62, no. 39, pp. 16101-16113, 2023.

[22] M. Montalti, A. Credi, L. Prodi, and M. T. Gandolfi, *Handbook of Photochemistry*, 3rd ed. Boca Raton: CRC Press, 2006, p. 664.
